# NEATmap: a high-efficiency deep learning approach for whole mouse brain neuronal activity trace mapping

**DOI:** 10.1093/nsr/nwae109

**Published:** 2024-03-26

**Authors:** Weijie Zheng, Huawei Mu, Zhiyi Chen, Jiajun Liu, Debin Xia, Yuxiao Cheng, Qi Jing, Pak-Ming Lau, Jin Tang, Guo-Qiang Bi, Feng Wu, Hao Wang

**Affiliations:** AHU-IAI AI Joint Laboratory, Anhui University, Hefei 230039, China; Anhui Province Key Laboratory of Biomedical Imaging and Intelligent Processing, Institute of Artificial Intelligence, Hefei Comprehensive National Science Center, Hefei 230088, China; National Engineering Laboratory for Brain-inspired Intelligence Technology and Application, School of Information Science and Technology, University of Science and Technology of China, Hefei 230026, China; Anhui Province Key Laboratory of Biomedical Imaging and Intelligent Processing, Institute of Artificial Intelligence, Hefei Comprehensive National Science Center, Hefei 230088, China; Division of Life Sciences and Medicine, University of Science and Technology of China, Hefei 230026, China; Anhui Province Key Laboratory of Biomedical Imaging and Intelligent Processing, Institute of Artificial Intelligence, Hefei Comprehensive National Science Center, Hefei 230088, China; National Engineering Laboratory for Brain-inspired Intelligence Technology and Application, School of Information Science and Technology, University of Science and Technology of China, Hefei 230026, China; Anhui Province Key Laboratory of Biomedical Imaging and Intelligent Processing, Institute of Artificial Intelligence, Hefei Comprehensive National Science Center, Hefei 230088, China; National Engineering Laboratory for Brain-inspired Intelligence Technology and Application, School of Information Science and Technology, University of Science and Technology of China, Hefei 230026, China; Anhui Province Key Laboratory of Biomedical Imaging and Intelligent Processing, Institute of Artificial Intelligence, Hefei Comprehensive National Science Center, Hefei 230088, China; National Engineering Laboratory for Brain-inspired Intelligence Technology and Application, School of Information Science and Technology, University of Science and Technology of China, Hefei 230026, China; Division of Life Sciences and Medicine, University of Science and Technology of China, Hefei 230026, China; Division of Life Sciences and Medicine, University of Science and Technology of China, Hefei 230026, China; Anhui Province Key Laboratory of Biomedical Imaging and Intelligent Processing, Institute of Artificial Intelligence, Hefei Comprehensive National Science Center, Hefei 230088, China; Division of Life Sciences and Medicine, University of Science and Technology of China, Hefei 230026, China; Interdisciplinary Center for Brain Information, Brain Cognition and Brain Disease Institute, Shenzhen-Hong Kong Institute of Brain Science-Shenzhen Fundamental Research Institutions, Shenzhen Institute of Advanced Technology, Chinese Academy of Sciences, Shenzhen 518055, China; AHU-IAI AI Joint Laboratory, Anhui University, Hefei 230039, China; Anhui Province Key Laboratory of Biomedical Imaging and Intelligent Processing, Institute of Artificial Intelligence, Hefei Comprehensive National Science Center, Hefei 230088, China; Anhui Province Key Laboratory of Biomedical Imaging and Intelligent Processing, Institute of Artificial Intelligence, Hefei Comprehensive National Science Center, Hefei 230088, China; Division of Life Sciences and Medicine, University of Science and Technology of China, Hefei 230026, China; Interdisciplinary Center for Brain Information, Brain Cognition and Brain Disease Institute, Shenzhen-Hong Kong Institute of Brain Science-Shenzhen Fundamental Research Institutions, Shenzhen Institute of Advanced Technology, Chinese Academy of Sciences, Shenzhen 518055, China; Anhui Province Key Laboratory of Biomedical Imaging and Intelligent Processing, Institute of Artificial Intelligence, Hefei Comprehensive National Science Center, Hefei 230088, China; National Engineering Laboratory for Brain-inspired Intelligence Technology and Application, School of Information Science and Technology, University of Science and Technology of China, Hefei 230026, China; Anhui Province Key Laboratory of Biomedical Imaging and Intelligent Processing, Institute of Artificial Intelligence, Hefei Comprehensive National Science Center, Hefei 230088, China; National Engineering Laboratory for Brain-inspired Intelligence Technology and Application, School of Information Science and Technology, University of Science and Technology of China, Hefei 230026, China

**Keywords:** whole-brain imaging, neuronal activity trace mapping, deep learning, quantitative analysis

## Abstract

Quantitative analysis of activated neurons in mouse brains by a specific stimulation is usually a primary step to locate the responsive neurons throughout the brain. However, it is challenging to comprehensively and consistently analyze the neuronal activity trace in whole brains of a large cohort of mice from many terabytes of volumetric imaging data. Here, we introduce NEATmap, a deep learning–based high-efficiency, high-precision and user-friendly software for whole-brain neuronal activity trace mapping by automated segmentation and quantitative analysis of immunofluorescence labeled c-Fos^+^ neurons. We applied NEATmap to study the brain-wide differentiated neuronal activation in response to physical and psychological stressors in cohorts of mice.

## INTRODUCTION

Quantitative analysis of the neuronal activity trace within hierarchical brain areas is crucial for the functional investigation of the brain. The neuronal activity-associated immediate early gene c-Fos is widely used to discover the responsive neuronal activation related to any specific stimuli [1]. Recently, with the development of versatile tissue clearing techniques [2–5] and high-speed volumetric imaging methods (e.g. light sheet microscopy) [6–10], comprehensive mapping of the whole-brain neuronal activity trace by counting activated cells has been demonstrated [11]. For whole-brain three-dimensional (3D) imaging, terabyte-scale image datasets can be easily collected, e.g. an adult mouse brain could be imaged in 1.5 hours with about a 4-terabyte dataset collected with a voxel size of $0.5 \times 0.5 \times 3.5\,\, {{\mu}}{{\rm {m}}^3}$ using our previously reported technique VISoR [12]. A dataset of this scale is quite challenging and tedious for most neuroscientists to analyze. Therefore, a user-friendly high-efficiency and quantitative analytical method is of particular importance for processing such whole-brain volumetric imaging datasets for the neuroscience field.

Recently, machine learning–backed algorithms for semantic segmentation of 3D images have been used for quantitative and automated analysis of large-scale volumetric imaging biomedical datasets [13–20], yielding exciting results. For whole-brain neuronal activity analysis, several methods have been reported, e.g. ClearMap [11] and CUBIC-Clound [5]. However, existing methods often have difficulties in achieving high accuracy and high efficiency at the same time for segmenting activated neurons in large cohort mice brains. Aside from segmenting the cells from several terabyte image datasets, a systematic analytical pipeline is also needed to illustrate the statistical differences among animals in different groups from various perspectives.

**Figure 1.**
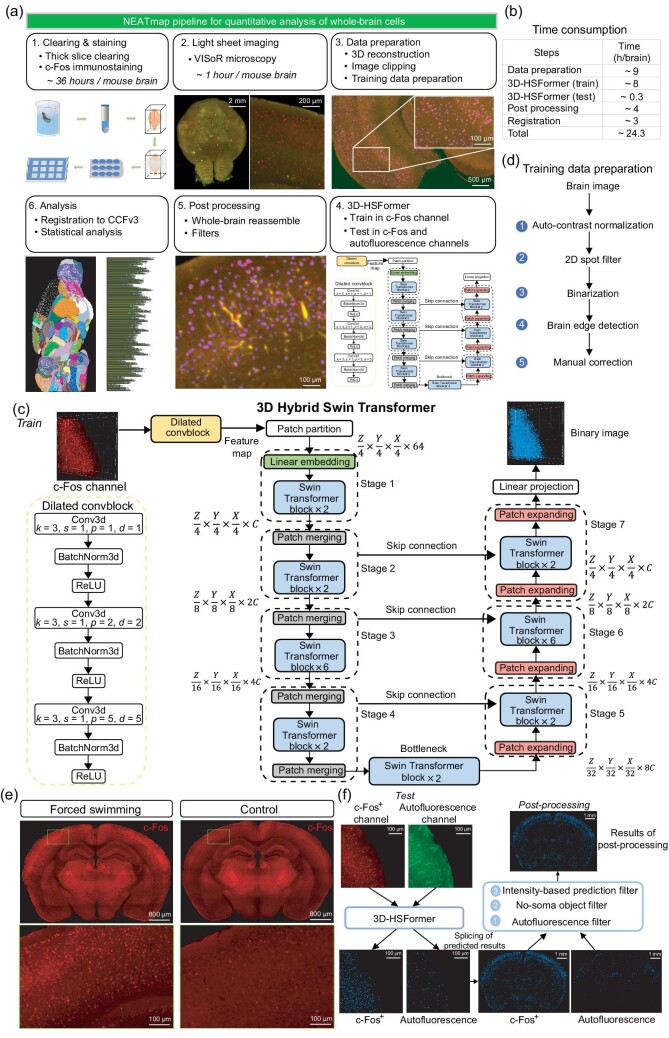
A pipeline for rapid clearing, immunostaining, imaging, cell segmentation and quantitative analysis of whole-brain neuronal activity. (a) Flow chart of the NEATmap pipeline. (b) Time consumption of each main step in the pipeline. (c) Three-dimensional hybrid Swin transformer architecturecomprising a dilated convolution block and a U-shape Swin transformer. (d) Processing of the training data preparation. (e) Virtual section (4 $\mu$m thick) of whole-brain c-Fos imaging by VISoR in the FST and the Ctrl groups. (f) Detailed pipeline of the 3D-HSFormer inferencing and post-processing of dual-channel whole-brain imaging datasets.

In this study, we propose NEATmap, a deep learning–based high-efficiency segmentation and analysis pipeline for whole-brain neuronal activity trace mapping. Our method consists of three main steps: (1) tissue clearing, immunostaining and high-speed volumetric imaging of mouse brains, as described in our previous study [12], allowing for high resolution dual-channel imaging of the c-Fos signal and autofluorescence of the tissue; (2) applying the 3D hybrid Swin transformer (3D-HSFormer) framework, designed for efficient automated brain-wide segmentation of activated neurons (completing in about 20 minutes); and (3) carrying out a comprehensive analysis of the neuronal activity trace within hierarchical brain structures of different levels with data and results registered to the Allen Common Coordinate Framework atlas (CCFv3), as well as individual variability in cohort animals and lateralized neuronal activation in two hemispheres (Fig. 1a and b and Table S1). To demonstrate the effectiveness and efficiency of this method, we applied the pipeline to whole-brain c-Fos imaging datasets of cohorts of mice tested using two different behavior paradigms. The performance of our pipeline outperforms ClearMap [11] and annotators using Ilastik [17]. Meanwhile, the neural network architecture we reported here outperforms many other artificial neural networks on different segmentation metrics.

## RESULTS

### 3D-HSFormer for automated whole-brain dual-channel segmentation

The Swin transformer [21] has outperformed many excellent convolutional neural networks (CNNs) in multiple computer vision tasks, while a larger training dataset was required because of a lack of *a priori* knowledge of CNNs. Here, we construct an artificial neural network named 3D-HSFormer, in which we combined a convolutional neural network with a transformer to segment cells in large volume three-dimensional image datasets (Fig. 1c). The input image is initially processed through a *dilated convblock* to obtain feature maps containing information at diverse levels. These feature maps are divided into various patches through *patch partition*. Each patch is treated as a ‘token’, resulting in the generation of a token sequence. Different *stages* composed of the core *Swin transformer blocks* are applied to extract the feature of the above token sequence. The extraction is done using a shifted window based on multi-head self-attention. The encoder of 3D-HSFormer comprises four *stages* for learning multi-scale features. The *bottleneck* still incorporates the *Swin transformer block* to capture deep feature representations. In the decoder, context features extracted from the three *stages* are fused through *skip connection* to restore the feature map resolution to the input resolution. The *linear projection* is used to obtain voxel-wise segmentation predictions from the above output results, and then the *SoftMax* function is utilized for obtaining the binary mask (Fig. S1 and Supplementary Methods).

A high-quality training dataset of immunostained c-Fos neurons with heterogeneous imaging profiles throughout the brain was needed to train a robust artificial neural network. Here, we generated the high-precision spot-like training masks [22] of c-Fos^+^ neurons with a segmentation-based approach (Fig. 1d, Fig. S2 and Supplementary Methods). The intensity threshold used for mask generation was determined by statistical analysis of the fluorescence intensity of immunostained neurons in several representative areas (Fig. 1e and Fig. S3). With this training dataset, 3D-HSFormer was trained for whole-brain image segmentation in about 8 hours using one GPU workstation. The trained network could be applied to the whole brain c-Fos^+^ cell segmentation in all other animals of the same batch without any further requirement of annotations.

The dual-channel whole-brain segmentation results in different patches were stitched back to the original brain slices separately. The segmentation results were post-processed with three filters: autofluorescence filter, no-soma object filter and intensity-based prediction filter (see the Materials and Methods section below), to exclude the non-neuronal results as much as possible (Fig. 1f and Video S1). After the post-processing, all the segmented neurons were aligned to the Allen mouse brain atlas [23] and then analyzed using hierarchical brain structures quantitatively.

### Performance evaluation of the 3D-HSFormer

**Figure 2.**
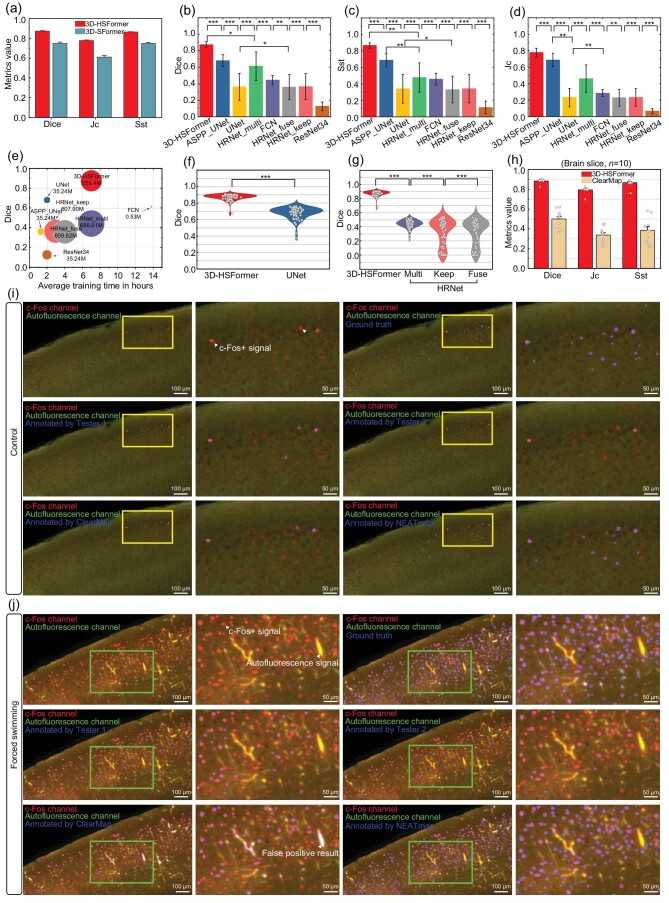
3D-HSFormer performance evaluation and comparison with other methods. (a) 3D-SFormer refers to 3D-HSFormer without the dilated convolution block in the ablation study. (b)–(d) Accuracy comparison among models Dice, Sst and Jc. For HRNet, HRNet_keep only outputs high-resolution features. HRNet_fuse fuses features of different scale resolutions. HRNet_multi forms a feature pyramid from the representation by fused output features. (e) The training efficiency of a model depends on its parameter size, training time and segmentation accuracy. The size of the parameters in the model is represented by the size of the bubble. (f) and (g) Comparison of 3D-HSFormer with UNet and HRNet in a brain slice (thickness 256 $\mu$m) by the metrics of Dice. The gray dots indicate 70 patches clipped from one brain slice. (h) Comparison of segmentation accuracy between 3D-HSFormer and ClearMap on 10 brain slices (thickness 256 $\mu$m). (i) and (j) Comparison of the c-Fos^+^ cell segmentation using 3D-HSFormer and other methods, including the ground truth annotated by three human experts, Ilastik used by two expert-level testers and ClearMap on the dual-channel data of the Ctrl group and FST group. Red represents the c-Fos channel. Green represents the autofluorescence channel. Blue represents the segmentation results. The Wilcoxon signed-rank test was used in (b)–(d), (f) and (g). ***P < 0.001, **P < 0.01, *P < 0.05; ns means no significance.

Convolutional local feature processing was applied to enhance the *a priori* knowledge of the transformer network [24]. We adopted this idea for our 3D-HSFormer. To validate the effectiveness of the architecture, we performed an ablation study. The result showed that 3D-HSFormer outperforms the 3D Swin transformer (3D-SFormer) architecture on multiple voxel-wise segmentation metrics (Fig. 2a).

To further evaluate the performance of 3D-HSFormer, we compared it with several baseline CNN [14,25–27] predictions. The ground truth for the test data was established through expert annotations (Fig. 2a–h). 3D-HSFormer outperformed other models on three voxel-wise segmentation metrics: the Dice coefficient (Dice), sensitivity (Sst) and the Jaccard coefficient (Jc) (Fig. 2b–d). All of these networks were trained with the same dataset prepared from an intact mouse brain (Fig. 2e), and tested with a dataset of a brain slice consisting of 70 patches of another mouse brain. Compared with existing segmentation models, Dice of 3D-HSFormer is significantly higher than the UNet and HRNet series. The segmentation performances of HRNet_keep and HRNet_fuse were not stable in different patches, indicating that the strategy using learning high-resolution feature maps and fusing multi-scale feature maps is not fully applicable to whole-brain neuronal activity trace segmentation (Fig 2f and g). These results indicate that the Swin transformer and the U-shaped network structure may play important roles in accurate segmentation (Fig. 2b–d). Therefore, 3D-HSFormer could accurately segment activated single neurons throughout the brain (Fig. S4).

We applied 3D-HSFormer to the brains of mice that undertook the forced swimming test (FST) and in the control (Ctrl) group (six animals in each group). In both the FST and Ctrl groups, 3D-HSFormer outperformed Ilastik and ClearMap not only with better segmentation accuracy, but also with higher efficiency (Fig. 2h–j and Table S2). The ground truth was established through annotation by an expert annotation and verified by two other experts. Two experienced technicians were assigned as testers to analyze the same dataset with Ilastik independently. Although the testers could avoid segmenting the autofluorescent non-neuronal structures with their extensive *a priori* knowledge, the segmentation results of both testers showed poorer accuracy in comparison to the result of 3D-HSFormer. Meanwhile, we noticed visible disparities between the results of two testers. This might be caused by the slight difference between the two testers on how they visually picked and manually annotated c-Fos^+^ cells as ground truth in Ilastik, and therefore got noteworthy differences in whole-brain c-Fos^+^ cell segmentation. The segmentation result of ClearMap showed improved accuracy in the c-Fos signal segmentation compared to the testers using Ilastik. We also noticed apparent non-c-Fos false positive structures such as autofluorescent blood vessels in the ClearMap segmentation results. These broadband autofluorescent structures could be filtered by analyzing an additional imaging channel of autofluorescence. 3D-HSFormer not only performed well at automated segmentation of activated neurons in the c-Fos channel, but was also good at segmenting other bright structures in the autofluorescence channel, indicating the strong transferability for inferencing the dataset with the features similar to the training dataset. Whether in the Ctrl group or the FST group, NEATmap demonstrates superiority compared to other methods in terms of performance assessed by various metrics (Fig. S5). Therefore, NEATmap can achieve more accurate detection of c-Fos^+^ neurons than other methods through dual-channel segmentation.

To demonstrate the transferability of NEATmap, we employed iDISCO cleared whole-brain image data for training on the 3D-HSFormer model. A comparison was made between the segmentation results of the whole-brain images and the outcomes produced by ClearMap. The findings indicate that 3D-HSFormer outperforms ClearMap across various segmentation metrics, with notably higher scores observed in the case of the Sst metric (Fig. S6). Additionally, we applied transfer learning to NEATmap for analyzing other cellular markers labeled volumetric datasets, including fluorescence *in situ* hybridization labeled somatostain (*Sst*), vesicular glutamate transporter 1 (*Vglut1*) and vesicular GABA transporter (*Vgat*) datasets. Experts labeled and verified ground truth datasets used for transfer learning of NEATmap on these cell-type marker imaging datasets. The segmentation results were also assessed by three metrics, demonstrating that NEATmap can be easily applied for other cell-type marker segmentations with high performance by small dataset transfer learning (Fig. S7).

### Whole-brain neuronal activity trace mapping of forced swimming tested mice

**Figure 3.**
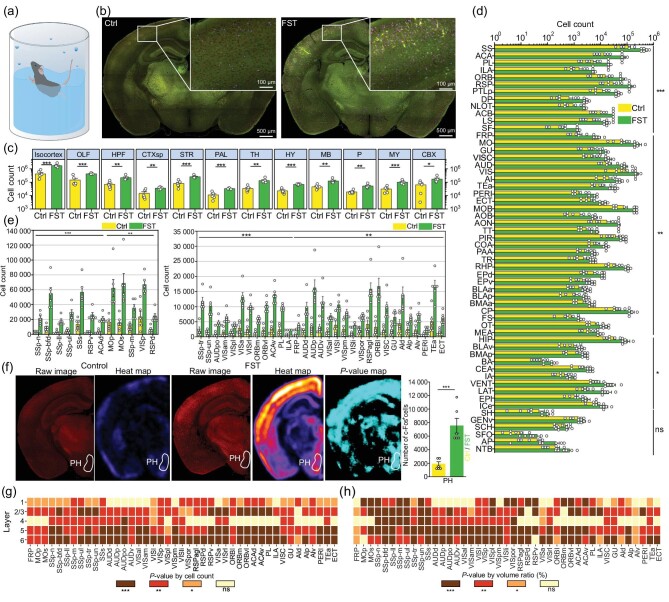
Whole-brain neuronal activity trace mapping of the forced swimming tested mice. (a) Schematic diagram of the forced swimming test. (b) The automated segmentation results of NEATmap in the Ctrl (left) and FST (right) groups. Red represents the c-Fos channel, green represents the autofluorescence channel, blue represents the segmented c-Fos^+^ cells. (c) Whole-brain c-Fos^+^ cell counting in the Ctrl and FST groups. (d) Automated segmentation of activated neurons by brain areas (level 6) sorted by adjusted *P*-values. Data are shown as the mean ± SE (*n* = 6). (e) Differential neuronal activation in multiple isocortical regions. Left panel: several brain areas have a large population of c-Fos^+^ cells. (f) The first and third columns are the projections of raw c-Fos signals in virtual sections (25 $\mu$m) of the FST and Ctrl mice. The second and fourth columns are c-Fos^+^ cell counting heatmaps of the FST and the Ctrl mice. The fifth column is an adjusted *P*-value map illustrating the differential neuronal activation in different brain areas in the FST-to-Ctrl comparison. (g) and (h) Layer-specific neuronal activity comparison between FST and Ctrl in the isocortex by cell counts and volume ratio (*n* = 6). The Student’s *t*-test was used in (c)–(h). ***P < 0.001, **P < 0.01, *P < 0.05; ns means no significance.

To systematically demonstrate the ability of NEATmap in analyzing the whole-brain neuronal activity trace in large cohort animals, we applied it to quantitatively elucidate the difference of neuronal activation in hierarchical brain areas between animals from the FST and Ctrl groups. The coordinates of the center of mass of each neuron in the segmentation results were mapped to the ABA CCFv3 (voxel size $25 \times 25 \times 25\, {{\mu}}{{\rm {m}}^3}$) through whole-brain alignment of our reconstructed mouse brain dataset (voxel size $4 \times 4 \times 4\, {{\mu}}{{\rm {m}}^3}$) (Fig. S8). With a first sight view of the segmentation results of 3D-HSFormer, significantly more neurons were activated in the cerebral cortex of mice in the FST group compared to the Ctrl group (Fig. 3a, b and Video S2). The total number of activated neurons in FST group mice were (4.0 ± 1.0) × 10^6^ (*n* = 6) (Table S3 and Video S3), which is significantly higher than in the Ctrl group ((1.2 ± 0.6) × 10^6^ (*n* = 6)).

In large structures, neuronal activation in the isocortex, olfactory (OLF) areas, striatum (STR), hypothalamus (HY) and medulla (MY) of FST mouse brains were significantly different from Ctrl animals (Fig. 3c). Among all 205 level-6 brain areas defined in ABA CCFv3, neuronal activation in many of them is significantly different in the FST group compared to the Ctrl group (Fig. 3d and Table S4). Within these areas, 55 of which have adjusted *P*-values (false discovery rate (FDR) *q*-values) <0.001, 102 of them have adjusted *P*-values <0.01, 31 have adjusted *P*-values <0.05 and 17 have no significant differences. Down to level-7 brain areas, c-Fos^+^ neurons show differential distribution in subregions of somatosensory (SS) and somatomotor (MO) areas. In subregions of visual and auditory areas, the activated neurons also show differential distribution, but the number is relatively low (Fig. 3e and Table S5).

Interestingly, neurons in the posterior hypothalamic (PH) nucleus, which have a role in regulating body temperature [28], were significantly activated in the FST group compared to the Ctrl group (Fig. 3f and Video S4), probably because of the low-temperature stimulation animals received during FST. Micrometer resolution 3D imaging, high-accuracy cell segmentation and registration enable us to perform the analysis more thoroughly (Video S5). Laminar cell count analysis revealed that the number of c-Fos positive neurons varies by layers and brain areas. Roughly, in most cortical areas, activated neurons are mainly found in layer 5 and layer 6, while neurons in layer 1 are relatively silent (Fig. 3g and Fig. S9c). We also quantified two other metrics, the volume ratio (Fig. S9a and d) and neuron density (Fig. S9b and e), and performed a layer-by-layer statistical hypothesis test (Fig. 3h) analysis for each subregion in the isocortex.

### Analysis of differential neuronal activation in FST mice

**Figure 4.**
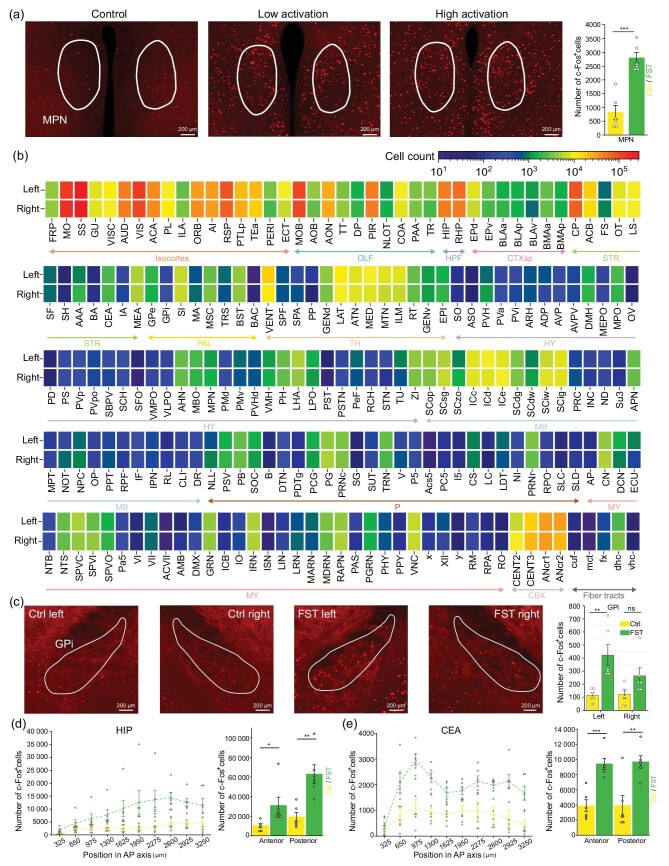
Analysis of differential neuronal activation in FST mice. (a) Varied neuronal activation in the MPN of different animals. Maximum-intensity projection of a 40-$\mu$m-thick virtual slice of the c-Fos signal. (b) The c-Fos^+^ cell counting of left and right hemispheric brain areas. (c) Themaximum-intensity projection (thickness 40 $\mu$m) of the c-Fos signal in the left and right GPis of Ctrl and FST animals. Right most panels: a significant difference can be observed in the left GPi, while no significant difference can be observed in the right half. (d) and (e) Differential neuronal activation along the anterior to posterior axis of the HIP and CEA. Data are shown as the mean ± SE (*n* = 6). The Student’s *t*-test was used in (a) and (c)–(e). ***P < 0.001, **P < 0.01, *P < 0.05; ns means no significance.

Differential neuronal activation in different animals coping with the same stimulation has been observed previously. Here in the forced swimming test, significantly higher neuronal activation in the medial preoptic nucleus (MPN), an area which plays a key role in body temperature control and stress response, was observed in the FST group compared to the Ctrl group. However, the number of c-Fos^+^ neurons was distinctive in each animal of the FST group. This may implicate the individual difference in animals’ neural activities when dealing with the same stimulation (Fig. 4a). Lateralization of the brain has long been a fascinating phenomenon in primates, and yet to be studied thoroughly in mice [29,30]. To address this question, we performed separate analysis of the neuronal activity trace in two hemispheres of every animal in the FST and Ctrl groups and found lateralized neural activity in many brain areas (Fig. 4b and Table S6). For the internal globus pallidus (GPi), an area related to motor control and motivation, there was significant difference in the number of c-Fos^+^ neurons between the FST group and the Ctrl group in the left hemisphere, while there was a nonsignificant difference in the right hemisphere (Fig. 4c). We found 26 brain areas with hemispheric significant to nonsignificant difference in the FST dataset (Table S6). We also analyzed the distribution of activated neurons along the anterior to posterior axis within two subcortical nuclei, the hippocampal (HIP) and central amygdala nucleus (CEA). The number of c-Fos^+^ neurons was more pronounced in the posterior HIP than in the anterior HIP (Fig. 4d). In contrast, the distribution of c-Fos^+^ neurons peaked in the anterior half of the CEA, but the total numbers were quite similar between the anterior and the posterior halves of the CEA (Fig. 4e).

### Systematic analysis of differential neuronal activation in the acute social defeat emotional stress model

**Figure 5.**
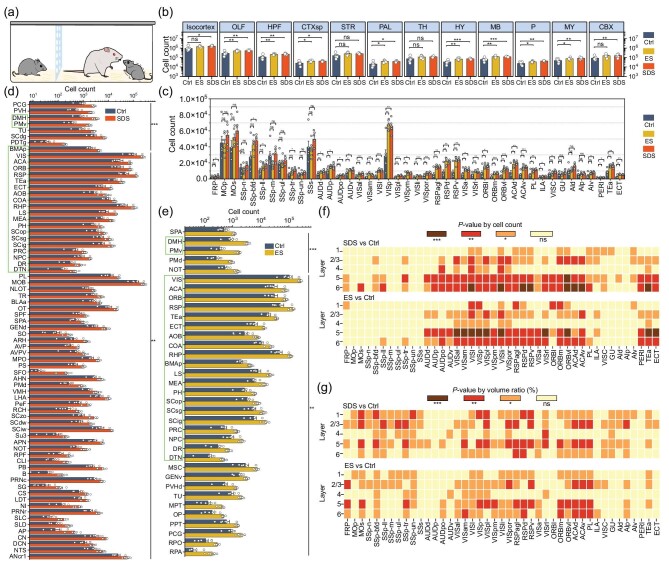
Whole-brain neuronal activity trace analysis of the multi-group acute social defeat and emotional stress model. (a) Schematic diagram of the acute social defeat emotional stress experiment. (b) Whole-brain c-Fos^+^ cells are counted in large brain areas (levels 3–5) in the SDS, ES and Ctrl groups. (c) Differential neuronal activation in subregions of the isocortex. (d) and (e) Automated segmentation of cell counts in brain areas of level 6 sorted by adjusted *P*-values in the SDS (d) and ES (e) groups. The green boxes show the common brain areas in the SDS and ES groups at the same significance level. Data are shown as the mean ± SE (*n* = 6). (f) and (g) Layer-specific neuronal activity comparison between SDS to Ctrl and ES to Ctrl in isocortical areas by cell counts and volume ratio (*n* = 6). The Student’s *t*-test was used in (b)–(g). ***P < 0.001, **P < 0.01, *P < 0.05; ns means no significance.

To further demonstrate the possibility of using NEATmap to illustrate the subtle difference between groups of animals in the same behavior paradigm, we used an acute social defeat emotional stress paradigm, in which an observer C57BL/6J mouse separated by a porous transparent plate would receive emotional stress (ES) without physical contact when the cohoused native C57BL/6J mouse received acute social defeat stress (SDS) from a novel CD1 mouse for 10 minutes (Fig. 5a). Then we applied NEATmap to analyze the brain-wide neuronal activity trace of animals among the SDS, ES and Ctrl groups. In the SDS group, neurons in the isocortex, OLF, hippocampal formation (HPF), cortical subplate (CTXsp), pallidum (PAL), HY, midbrain (MB), pons (P), MY and cerebellar cortex (CBX) showed significantly higher activation than in the Ctrl group. However, in the ES group, c-Fos^+^ neurons in many of the above areas also showed significant differences compared to the Ctrl group: OLF, HPF, CTXsp, PAL, HY, MB, P and MY, but not in the isocortex and CBX (Fig. 5b). We looked deeper into the isocortex, and not surprisingly found that the ES group has relatively low-level neuronal activation in somatosensory areas and relative high-level neural activity in the visual cortex than the SDS group in comparisons with the Ctrl group (Fig. 5c and Fig. S10c). Systematic analysis of the neuronal activations based on automated segmentation, atlas registration, cell counting and statistical quantification in level-6 brain areas (205 in total) revealed significant differences in the c-Fos^+^ cells in 137 and 90 brain areas in the SDS and ES groups compared to the Ctrl group, respectively (Table S7). The c-Fos^+^ neurons in the dorsomedial nucleus of the hypothalamus (DMH) and the ventral premammillary nucleus (PMv) regions exhibited the same high-level significance (*P* < 0.001) in both the SDS and ES groups compared to the Ctrl group. At a significance level of *P* < 0.01, 20 more common regions were found in both groups (Fig. 5d and e). We also did laminar analysis with this experiment. Significant differences were observed in layers 5 and 6 of the auditory, visual and posterior cortical regions in the SDS and ES groups compared to the Ctrl group (Fig. 5f). The volume ratio and cell density of activated neurons in the isocortex were analyzed in all three groups (Fig. S10a and b). It can be observed that, compared to the ES group, the SDS group had multiple layered areas that were significantly different from the Ctrl group in terms of volume ratio indicators (Fig. 5g and Fig. S10d). In the laminar cell density analysis, the SDS group and the ES group had a higher density of c-Fos^+^ cells in layers 4 and 5 of the visual areas (Fig. S10e).

### Animal individual variability and correlation analysis of neuronal activation in thalamic and hypothalamic areas

**Figure 6.**
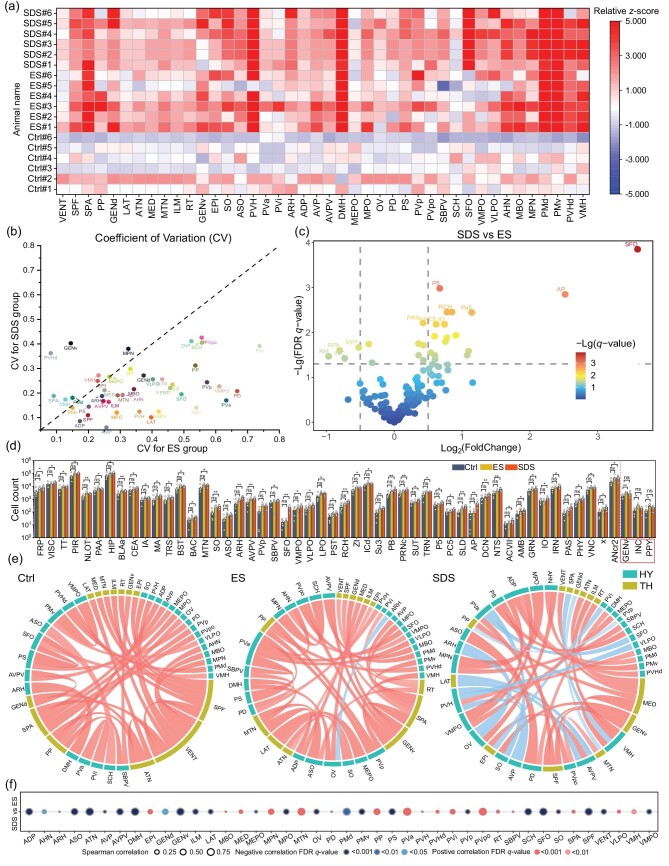
Animal individual variability and correlation analysis of neuronal activation in thalamic and hypothalamic areas. (a) Relative *z*-score plot of 42 brain areas of 18 animals from three groups. (b) Coefficient variation plot of 42 brain areas between the SDS and ES groups (*n* = 6). (c) Comparisonof differential neuronal activation by direct (SDS) and indirect (ES) stressors. The two vertical lines represent the fold change in c-Fos^+^ cells <−1.4 and >1.4, respectively, and the horizontal line represents the FDR *q*-value of 0.05. The color of the dots represents the level of the FDR *q*-value. (d) Specific brain areas with significant neuronal activation induced by direct stressor or indirect stressor only. The red box indicates areas that only have significant differences between the ES group and Ctrl group but not in the SDS group versus the Ctrl group, and the remaining areas have significant differences to the opposite condition. (e) The correlation of c-Fos^+^ cell counts in the Ctrl, ES and SDS groups across 42 thalamic and hypothalamic areas. The highest correlation is plotted for each area by the maximum absolute value. The red lines indicate positive correlation, while the blue lines indicate negative correlation. (f) Spearman correlation analysis of c-Fos^+^ cells in 42 brain areas between the SDS and ES groups. The size of the bubbles represents the correlation levels, while the color of the bubbles represents the FDR *q*-value levels. Red indicates positive correlation, while blue indicates negative correlation. The Student’s *t*-test was used in (c), (d) and (f). ***P < 0.001, **P < 0.01, *P < 0.05; ns means no significance.

Individual variability is an important effect factor that has been paid a lot attention in biomedical studies. Here with whole-brain imaging and segmentation, we could address this issue more systematically. We plotted the relative *z*-score (see the Materials and Methods section below) and the coefficient of variation (CV) of the SDS group versus the ES group of the thalamic and hypothalamic brain areas (Fig. 6a, b and Table S8). The results revealed that the ES group showed higher individual difference in the relative *z*-score plot and higher CV performance in many brain areas compared to the SDS group.

To further investigate the differential neuronal activation induced by direct and indirect stressors in different brain areas between the SDS and ES groups, we created a volcano plot of the fold change and adjusted *P*-values. Six brain areas exhibited significantly higher neuronal activation in the SDS group versus the ES group (fold change >1.4 and adjusted *P*-value <0.01), including the subfornical organ (SFO), area postrema (AP), peritrigeminal zone (P5), paratrigeminal nucleus (Pa5), retrochiasmatic area (RCH), inferior olivary complex (IO) and parvicellular motor 5 nucleus (PC5) (Fig. 6c). SFO plays a key role in cardiovascular regulation and energy homeostasis [31]. RCH is associated with the regulation of the circadian rhythms, including hormone production, body temperature, etc. [32]. AP contains sensory neurons that detect circulating chemical messengers in the blood and transduce them into neural signals [33]. P5, Pa5 and PC5 are parts of the trigeminal nerve, where the activity of sensory and motor neurons may reflect the physical interaction during the social defeat experiment [34]. IO is functionally related to motor coordination and sensorimotor integration [35]. Significantly different neuronal activations in these areas imply that physical interaction such as fighting requires much higher neural regulation of basic physiological functions. The mechanisms underlying these findings need to be further investigated thoroughly. When compared to the Ctrl group, we identified 50 brain areas with significant difference in the SDS group and nonsignificant difference in the ES group. Meanwhile, three brain areas exhibited significant differences in the ES group and nonsignificant differences in the SDS group compared to the Ctrl group (Fig. 6d).

Since the brain is a highly innervated network, none of the brain areas is isolated. So, the correlation between the neuronal activation of different brain areas is a valuable metric to find intriguing connections. We attempted to assess the correlation of brain activity between regions using the Spearman correlation coefficient ρ (detailed in the Materials and Methods section below). For thalamic and hypothalamic areas, positive correlations (0 < ρ < 1) were observed between brain areas within the Ctrl group. In the ES group, a negative correlation (−1 < ρ < 0) was found between the arcuate hypothalamic nucleus (ARH) and vascular organ of the lamina terminalis (OV). In the SDS group, more brain areas showed negative correlations in between (Fig. 6e). The correlations between different brain areas within separate groups indicate that the interplay between these regions is modulated by various stressors in different groups. A positive correlation between brain areas suggests synchronization or coordination, while a negative correlation implies a relationship where the activation of one brain area is associated with the deactivation of another. In the SDS group, negative correlations were observed between multiple brain area pairs, suggesting distinct responses of these brain areas to this stressor. Therefore, there might be direct or indirect inhibitory connections between these brain areas. To further investigate the difference in brain areas between the SDS and ES groups, we conducted a correlation analysis of c-Fos^+^ neurons in 42 thalamic and hypothalamic areas. Of these, 25 brain areas showed significant negative correction between the two groups (Fig. 6f).

## DISCUSSION

The application of deep learning algorithms in biomedical imaging data related tasks has been explored intensively since the renaissance of artificial neural networks [36,37]. NEATmap is designed and built as a deep learning–based high-efficiency open-source software to facilitate the segmentation and systematic analysis of the whole-brain neuronal activity trace for the neuroscience field. In this study, combined with high-speed, high-resolution light sheet imaging, we applied NEATmap to analyze the differential neuronal activity in hierarchical brain areas from brain lobes to cortical layers in forced swimming and acute social defeat emotional stress behavioral models. Besides, lateralized differences of neuronal activations were revealed in the same brain areas between two hemispheres. The individual variability in a large cohort of animals was analyzed as well. These results suggest that our pipeline can be efficiently used for the brain-wide neuronal activity trace analysis of a large cohort of animals of various behavioral tests with multiple groups.

The combination of immunostaining-compatible tissue clearing techniques with light sheet microscopy has enabled high-throughput whole-brain neuronal activity trace mapping. However, due to the centimeter-scale mouse brain size, the efficiency and resolution are therefore hardly reconciled [38]. Typically, during the sample preparation, whole-mount brain tissue clearing and immunostaining usually need several weeks, e.g. 18 days in ClearMap (iDISCO+) [2] and 2–9 weeks in CUBIC-Clound (CUBIC-HV protocol, version 1.0) [5]. In contrast, with the whole brain sliced into 300-$\mu$m-thick continuous sections, the time for sample preparation is usually no more than three days, including the time for fixation, embedding, slicing, clearing and immunostaining (Table S1 and Fig. S11). For whole-mount mouse brain light sheet fluorescence microscopy imaging, the voxel size was $6.45 \times 6.45 \times 7.0\,\,\mu{\rm {m}}^3$ in the CUBIC-Clound pipeline, and 4 × 4 × 3 $\mu{\rm {m}}^3$ in the ClearMap pipeline with a brain hemisphere imaged in an hour. The c-Fos^+^ cell nuclei are only a few voxels in datasets acquired at these resolutions, and are difficult to detect with high accuracy. Here in the NEATmap pipeline, with high-speed, high-resolution imaging of VISoR, a whole brain can be imaged in half an hour with a voxel size of 1 × 1 × 3.5 $\mu{\rm {m}}^3$, which makes cell detection much more accurate.

NEATmap enables more accurate 3D segmentation of c-Fos^+^ (Fig. 2i and j) by automated inferencing of the dual-channel whole-brain imaging dataset in about 40 minutes with a high-performance GPU card (V100S-32GB). Beside the efficiency, existing analysis tools often struggle to distinguish false-positive signals if there is no human intervention, resulting a relative low accuracy. In NEATmap, high efficiency and high accuracy are accomplished through the 3D-HSFormer deep learning architecture and an optimal analysis pipeline. Instead of imaging the c-Fos channel only, we proposed adding an imaging channel for autofluorescence, which has proved dramatically helpful in removing the non-cell segmentation results with similar features to c-Fos^+^ cells. Data of two channels are input to the 3D-HSFormer for segmenting cell-like objects. NEATmap uses the segmentation results from the autofluorescence channel as reference to remove the false-positive segmentation signals in the c-Fos channel with a few post-processing steps. A simple justification of c-Fos immunostaining intensity is included as a parameter for signal intensity-based filtering of datasets collected through different c-Fos labeling protocols and imaging methods during the post-processing step. The core of the NEATmap pipeline is the deep learning–based 3D segmentation model, which is a main affecting factor for the accuracy and efficiency of the whole analysis pipeline. Here in our study, the 3D-HSFormer can be continuously updated or replaced with other future models. There is a trade-off between the size of subvolume and the batch size while considering the model parameters and the memory size of GPUs.

With the advancement of computation power, we believe that neural networks will be allowed to input larger size data and more batches. This will further improve the efficiency and accuracy of automated whole-brain segmentation.

In summary, we present a simple-to-adopt pipeline and software to systematically analyze the whole-brain neuronal activity in a large cohort of animals. With our optimized sample preparation and imaging methods, whole-brain neuronal activity trace mapping can be completed in a few days. We expect NEATmap to be widely used in different studies, e.g. neuronal activity in response to specific stimulation, intrinsic or homeostatic neuronal activity, drug screening, etc.

## MATERIALS AND METHODS

For a more detailed description of the materials and methods employed in this study, we refer the reader to the online supplementary material.

### Animals

We used 8–12 weeks old male C57BL/6J mice for this study. All animals were purchased from Shanghai-Slac laboratory animal co., Ltd. All animals used in this study were group housed with 12/12-hours light/dark cycle (light on at 7 a.m.). Food and water were provided *ad libitum*. Animals were group housed for a week before experiment. All animal experiments were carried out following the protocol (ABSL-2-2022030402) approved by the Animal Care and Use Committee of the institute.

### Behavior experiments

For the forced swimming test, the animal was put into a 5-L glass beaker with half-filled water at 25$\, ^\circ$C for 5 minutes. The animal’s behavior was recorded with a video camera (Video S6). For the acute social defeat emotional stress test, the C57BL/6J mice were cohoused by pairs for a week before experiment. Retired male CD1 mice were screened for the following social defeat experiment.

### Sample preparation

The sample preparation pipeline is described in Fig. S11.

### Whole-brain light sheet microscopic imaging

The refractive index-matched brain slice was transferred to a VISoR imaging system, described in previous work [39]. Briefly, the system was equipped with four lasers (Coherent, OBIS series) and a sCMOS camera (Hamamatsu, Flash 4.0 v3). All images were collected through a 10× 0.3 NA water-immersion objective lens (Olympus) and a 0.63× adaptor (TV0.63, Olympus). The final voxel size was about 1 × 1 × 3.5 ${{\mu }}{{\rm {m}}^3}$. The c-Fos signal was collected at the 561-nm channel (Video S7); meanwhile, the 488-nm channel was also collected for autofluorescence correction.

### 3D-HSFormer network design

The *linear embedding* maps the input features to any dimension linearly. The *Swin transformer block* consists of stacked *patch merging* layers and *patch expanding* layers according to the parameters {2, 2, 6, 2} in the encoder and decoder, respectively. *Patch merging* and *patch expanding* represent down-sampling and up-sampling at the encoder and decoder, respectively. The *skip connection* refers to fusing the multi-scale features of the encoder with the features up-sampled in the decoder. The fusion of the shallow features of the encoder and the deep features of the decoder can prevent losing the down-sampled feature information [25]. The *linear projection* is used to linearly map the up-sampled features.

### Statistics

The statistical significance of *P*-values was obtained using the Student’s *t*-test, and the FDR *q*-value was used to control for false detection rates for multiple comparisons, with sample size (*n*), as shown in the main text and the supplementary figure legends. The statistical tests mentioned above were implemented using the SciPy library https://scipy.org/ in Python. The Benjamini–Hochberg multiple comparison test [40] was used to control the FDR.

## Supplementary Material

nwae109_Supplemental_Files

## Data Availability

The openly available c-Fos whole-mount immunostaining data obtained with iDISCO can be found at https://osf.io/5qzn7/. The total size of the mouse whole-brain dual-channel VISoR imaging datasets (both raw and processed) of this study exceeded 10 terabytes. Uploading it to a public data repository is impractical. We prepared an instance dataset of a mouse whole-brain dual-channel imaging for validating the automated segmentation results of NEATmap, including Fig. 1a and Videos S1 and S2 of dual-channel brain slices and segmentation results, as well as high-resolution mouse brain slices showing c-Fos^+^ cells in Fig. 1e (available at https://zenodo.org/record/8133486).

## CODE AVAILABILITY

The source code for all modules of NEATmap was implemented in Python and has been deposited in a public online repository (https://github.com/mesobrain/NEATmap_code). Software for NEATmap can be found in the NEATmap GitHub repository: https://github.com/mesobrain/NEATmap_software.
