# Supplementary material for: NEATmap: a high-efficiency deep learning approach for whole mouse brain neuronal activity trace mapping
Source: Natl Sci Rev. 2024 Mar 26;11(5):nwae109. doi: 10.1093/nsr/nwae109 (PMC11145917; doi:10.1093/nsr/nwae109)
Supplement: nwae109_Supplemental_Files [file nwae109_supplemental_files.zip › Supplementary_figure_2.pdf]

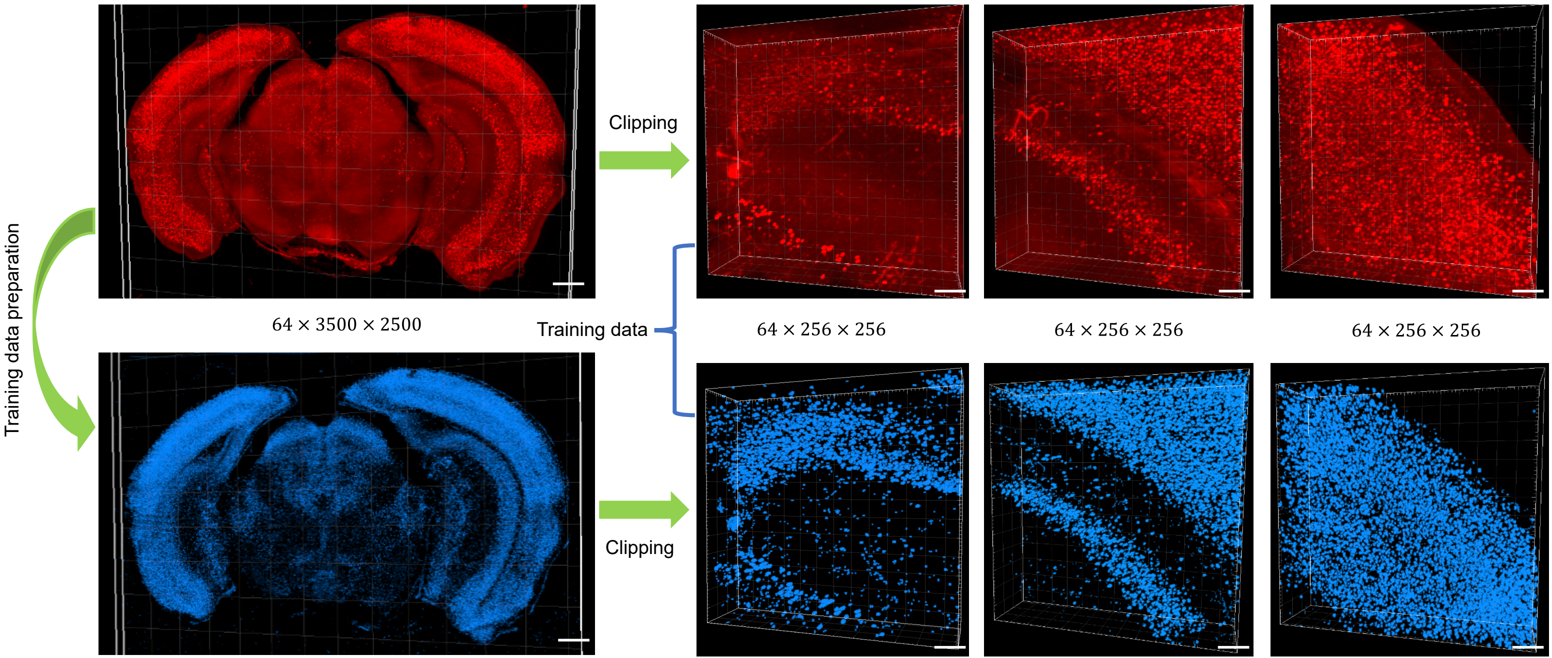
Training data preparation
Clipping
64 × 3500 × 2500
Training data
64 × 256 × 256
64 × 256 × 256
64 × 256 × 256
Clipping
