# Supplementary material for: NEATmap: a high-efficiency deep learning approach for whole mouse brain neuronal activity trace mapping
Source: Natl Sci Rev. 2024 Mar 26;11(5):nwae109. doi: 10.1093/nsr/nwae109 (PMC11145917; doi:10.1093/nsr/nwae109)
Supplement: nwae109_Supplemental_Files [file nwae109_supplemental_files.zip › Supplementary-r.pdf]

NEATmap: a high-efficiency deep learning approach for whole mouse brain neuronal activity trace mapping

Weijie Zheng[1,2,3,†], Huawei Mu[2,4,†], Zhiyi Chen[2,3], Jiajun Liu[2,3], Debin Xia[2,3], Yuxiao Cheng[4], Qi Jing[4], Pak-Ming Lau[2,4,5], Jin Tang[1,2,*], Guo-Qiang Bi[2,4,5,*], Feng Wu[2,3,*] and Hao Wang[2,3,*,‡]

[1] AHU-IAI AI Joint Laboratory, Anhui University, Hefei, 230039, Anhui, China.

[2] Anhui Province Key Laboratory of Biomedical Imaging and Intelligent Processing, Institute of Artificial Intelligence, Hefei Comprehensive National Science Center, Hefei, 230088, Anhui, China.

[3] National Engineering Laboratory for Brain-inspired Intelligence Technology and Application, School of Information Science and Technology, University of Science and Technology of China, Hefei, 230026, Anhui, China.

[4] Division of Life Sciences and Medicine, University of Science and Technology of China, Hefei, 230026, Anhui, China.

[5] Interdisciplinary Center for Brain Information, Brain Cognition and Brain Disease Institute, Shenzhen-Hong Kong Institute of Brain Science-Shenzhen Fundamental Research Institutions, Shenzhen Institute of Advanced Technology, Chinese Academy of Sciences, Shenzhen, 518055, China.

* Corresponding author(s). E-mail(s): J. Tang (tangjin@ahu.edu.cn); GQ. Bi (gqbi@ustc.edu.cn); F. Wu (fengwu@ustc.edu.cn) and H. Wang (haowang@ustc.edu.cn)

[†] These authors contributed equally to this work.

[‡] Lead contact.

Methods

Animals

8-12 weeks old male C57BL/6J mice were used for this study. All animals were purchased from Shanghai-Slac co., ltd. All animal used in this study were group housed with 12/12-hours light/dark cycle (light on at 7 a.m.). The food and water were provided ad libitum. Animals were group-housed for a week before experiment. All animal experiments were carried out following the protocol (No. ABSL-2-2022030402) approved by the Animal Care and Use Committee of the institute.

Behavior experiments

For forced swimming test, animal was put into a 5 L glass beaker with half-filled water at 25 °C for 5 minutes. Animal behavior was recorded with a video camera (Supplementary Video 6). After swimming the animal was dried with cotton tissue and a nearby heater before returning back to the home-cage for rest. The animal was sacrificed with transcardial perfusion for brain collection 85 minutes later after swimming. For acute social defeat emotional stress test, the C57BL/6J mice were cohoused by pairs for a week before experiment. Retired male CD1 mice were screened for following social defeat experiment. The social defeat stress (SDS) mouse was subjected to a novel CD1 mouse for 10 minutes while the cohoused emotional stress (ES) mouse receiving indirect stimulation by observing the SDS mouse in the same chamber. ES mice were isolated by a porous transparent during the test to the avoid having physical contact. After the stress test, mice were put back and rested in home-cage for 80 minutes before been sacrificed for whole brain neural activity mapping.

Sample preparation

Sample collection. The sample preparation pipeline is described in Supplementary Fig. 11. After the behavior test, all animal was anesthetized intraperitoneal injection of 1% pentobarbital sodium (80 mg/kg). All brains were collected by transcardial perfusion of 40 mL 1x phosphate salient buffer (PBS) and 20 mL 4% paraformaldehyde (PFA) respectively. Then were immersed in 4% PFA at 4 °C overnight for further fixation.

Embedding. The brain was immersed in hot 10% gelatin solution in a small plastic cube, then the cube was moved into ice cold water bath for gelatin clotting. Then the embedded sample was put back into 4% PFA for 24 hours' further fixation.

Slicing. All brains were sliced into 300 μm thick brain sections by a homemade vibrational slicer. All sections of each brain were collected sequentially.

Immunostaining. The slices were cleared with 0.5% Triton-X100 in PBS with gentle shaking at 37 °C for 6 hours. Then the slices were immune-stained with c-Fos (Cell Signaling Technology, #2250, 1:1500 dilution) primary antibody and anti-Rabbit secondary antibody (JacksonImmunoResearch, #711-165-152, 1:500 dilution).

Slice mounting and refractive index matching. Slices of each brain were mounted on a customized glass slide $(100\,mm \times 100\,mm)$ for the convenience of imaging. The sample slide was transferred into refractive index matching solution (RIMS) over night for homogenizing the optical property of the slice and surrounding solution.

Whole brain light sheet microscopic imaging

The refractive index-matched brain slices were transferred to a VISoR imaging system described as previously [1]. Briefly, the system was equipped with four lasers (Coherent, OBIS series) and a sCMOS camera (Hamamatsu, Flash 4.0 v3). All images were collected through a 10x 0.3 NA water-immersion objective lens (Olympus) and a 0.63x adaptor (TV0.63, Olympus). The final voxel size was about $1 \times 1 \times 3.5\,\mu m^3$. The c-Fos signal was collected in 561 nm channel (Supplementary Video 7), meanwhile, the autofluorescence in 488 nm channel was also collected for non-neuronal structure detection.

Whole brain image reconstruction

The reconstruction process [2] is divided into five steps. (1) According to the real coordinates of the captured images, they are stacked into columns. Whole slices were reconstructed from columns by the correction coefficients and true coordinates of the overlapping regions of each column. (2) The upper and lower surfaces of each contrast-enhanced brain slice were fitted by linear regression and interpolation. (3) The rigid transformation and B-spline [3] algorithm was used to detect the texture and edges of opposing surfaces of adjacent brain slices. (4) Constraints of the adjacent correspondence displacement and displacement vector to prevent the accumulation and propagation of multiple slip errors. (5) The distortion of slice size and shape was minimized by using moving-least-square [4].

Training data preparation

We generate the training dataset through the following five steps.

1. Auto-contrast normalization. Due to the large variability of signal intensities within and between brain slices, the mean and standard deviation of the intensities were first fitted to a Gaussian distribution to the whole stack intensity profile.

Intensity filter. The intensity interval of stretch was obtained from the mean and standard deviation of the fitted Gaussian distribution. In the Eq. (1), $\min_{stre}$ and $\max_{stre}$ are the min and max of the filtered image, $\min_{raw}$ and $\max_{raw}$ are min and max of the raw image, mean and std are the mean and standard deviation of the Gaussian distribution, λ and γ are scale factors. The smaller the values of λ and γ are, the higher the contrast will be. x is the pixel intensity in the Eq. (2).

$$\min_{stre} = \max(mean - \lambda \times std,\ \min_{raw})$$

$$\max_{stre} = \min(mean + \gamma \times std,\ \max_{raw}) \quad (1)$$

$$f(x) = \begin{cases} \max_{stre}, x > \max_{stre} \\ \min_{stre}, x \le \min_{stre} \end{cases} \quad (2)$$

Normalization of intensities. The raw image intensities are normalized to a range of 0 to 1.

$$N(x) = \frac{x - \min_{stre}}{\max_{stre} - \min_{stre}} \quad (3)$$

2. 2D spot filter. For strongly supervised training on the transformer architecture, we need to perform spot segmentation of c-Fos^{+} punctate cells. The 2D/3D spot filter calculates the Laplacian of Gaussian on the image in 2D/3D, respectively, both of them can detect the major structures in the 3D image stack. However, subtle differences have been observed when applied to 3D cell imaging datasets [5]. For the c-Fos expressed cells in each 2D frame there are more general spot-like morphological structures, rather than individual circular structures. 3D spot filter may fail to detect the complete structure of cells, while 2D spot filter showing a better detection completeness (Supplementary Fig. 12). Therefore, we used 2D spot filter to conduct preliminary detection of c-Fos^{+} signal in the Training data preparation process (Fig. 2d).

3. Binarization. The step converts the 3D detection results of c-Fos^{+} from the 2D spot filter into 3D mask annotations.

4. Brain edge detection. There are some cells with stronger signal intensity at the border of the brain slice, which are not the result of c-Fos immunofluorescence signal. We detect brain boundaries using the method of Canny edge detection [6] to remove them.

5. Mannel correction. The results obtained above were checked and recomputed with optimized parameters. The high-precision masks generated through the aforementioned process, combined with the image data, constitute the training dataset (Supplementary Figure 2).

3D-HSFormer architecture

We have designed a hybrid architecture artificial neural network with dilated convolutional neural network and transformer (Fig. 1c) for segmenting dual-channel (c-Fos and autofluorescence channels) whole-brain imaging datasets. The hybrid architecture outperformed the pure transformer architecture [7]. Dilated convolution [8] can obtain information at different scales and increase the receptive field without reducing the image resolution. The dilation rate (d) is set to $\{1, 2, 5\}$ in the *Dilated convblock* (Fig. 1c), respectively. The Swin Transformer architecture [9] showed great performance in tasks such as classification, object detection, and medical image segmentation [10] in 2D images. We further extended it to three dimensional for segmenting small and dense targets (e.g., c-Fos^{+} cells in whole mouse brain) in 3D microscopic images.

The proposed transformer architecture is mainly divided into encoder and decoder structures. The encoder is used to learn the features of different scales' output of the dilated convolution block, and the

decoder is utilized to up-sample the learned features to obtain the segmentation of each c-Fos[+] cell. We described the modules in the encoder and decoder in details as following.

Encoder. Assume that the input image size is $Z \times Y \times X \times 1$, the feature map size obtained after the *Dilated convblock* is $Z \times Y \times X \times 16$. The *Patch partition* can divide the feature map into non-overlapping patches of size $\frac{Z}{4} \times \frac{Y}{4} \times \frac{X}{4} \times 64$ with a patch size of $4 \times 4 \times 4$. The *Linear embedding* maps the input features to any dimension (denoted as C) linearly. The *Swin Transformer block* mainly consists of LayerNorm (LN) layer, multi-head attention module, residual connection and 2-layer multi-layer perceptron (MLP) [9]. Among them, two successive *Swin Transformer blocks* use the window-based multi-head self-attention (W-MSA) module and the shifted window-based multi-head self-attention (SW-MSA) (Supplementary Fig. 1b). The above two successive *Swin Transformer blocks* can be formulated as:

$$\hat{z}^{l} = W\text{-}MSA\left(LN(z^{l-1})\right) + z^{l-1} \tag{4}$$

$$z^{l} = MLP\left(LN(\hat{z}^{l})\right) + \hat{z}^{l} \tag{5}$$

$$\hat{z}^{l+1} = SW\text{-}MSA\left(LN(z^{l})\right) + z^{l} \tag{6}$$

$$z^{l+1} = MLP\left(LN(\hat{z}^{l+1})\right) + \hat{z}^{l+1} \tag{7}$$

where $\hat{z}^{l}$ and z^{l} refer to the output features of the (S)W-MSA module and the MLP module of the lth block, respectively. As reported in previous works [7, 9], self-attention is calculated as follows:

$$Attention(Q, K, V) = SoftMax\left(\frac{QK^{T}}{\sqrt{d}} + B\right)V \tag{8}$$

where Q, K, $V \in \mathbb{R}^{M^{3} \times d}$ indicate the query, key and value matrices; M^{3} is the number of patches in a window, and d is the query or key dimension. And, the values in B are taken from the bias matrix $\hat{B} \in \mathbb{R}^{(2M-1)\times(2M-1)\times(2M-1)}$.

Linear embedding and two successive *Swin Transformer blocks* form *Stage 1*. The Swin Transformer block cannot change the dimension of the token sequence. *Patch merging* consists of *Stage 2* with two successive *Swin Transformer blocks*, and *Stage 3* with six successive *Swin Transformer blocks*. *Patch merging* involves down-sampling the token by $2\times$, resulting in a feature dimension that is $2\times$ the original size. *Stage 4* comprises two *Patch merging* layers followed by two successive *Swin Transformer blocks*, which output deeper feature. A *Bottleneck*, formed by two successive *Swin Transformer blocks*, is employed to learn deep features.

Decoder. *Patch expanding* involves up-sampling the token by $2\times$. For instance, in the *Bottleneck*, features are passed to the first *Patch expanding* layer. This layer initially increases the feature dimension ($\frac{Z}{32} \times \frac{Y}{32} \times \frac{X}{32} \times 16C$) to $2\times$ the original size ($\frac{Z}{32} \times \frac{Y}{32} \times \frac{X}{32} \times 8C$) using a linear layer. Subsequently, a

rearrange operation is employed to $2\times$ the input feature resolution and reduce the dimension to quarter of the original ($\frac{Z}{32}\times\frac{Y}{32}\times\frac{X}{32}\times 16C \rightarrow \frac{Z}{16}\times\frac{Y}{16}\times\frac{X}{16}\times 4C$). *Patch expanding* consists of *Stage 5* and *Stage 6* with two successive *Swin Transformer blocks* and six successive *Swin Transformer blocks* respectively. *Stage 7* comprises two *Patch expanding* layers followed by two successive *Swin Transformer blocks*. It's worth mentioning that the final *Patch expanding* in *Stage 7* involves an $4\times$ up-sampling. The *Skip connection* refers to fusing the multi-scale features of the encoder with the features up-sampled in the decoder. The fusion of the shallow features of the encoder and the deep features of the decoder can prevent losing the down-sampled feature information [11]. The *Linear projection* is used to linearly map the up-sampled features.

Training schedule

The network was trained and tested on a workstation equipped an NVIDIA Tesla V100S-PCIe with 32GB RAM. The network was developed with Python 3.6 and Pytorch 1.6.0 [12]. The images and labels in the training data were prepared from 3D images of the brain slices and training data preparation results by clipping into subvolumes, the size of which is $64\times256\times256$ pixels (Supplementary Fig. 2). The network training was driven by a Stochastic Gradient Descent (SGD) optimizer with a learning rate of 1×10^{-2} and a weight decay rate of 1×10^{-4} for the moment 0.99. In the training phase, we used the data of a whole brain in the FST group, set the batch size to 2, and trained the network for 10 epochs. The total training loss function is divided into two parts: cross-entropy loss and dice loss [13]. By using this loss combination, the frequent occurrence of a certain class due to clipping can be prevented. The cross-entropy loss and dice loss are calculated as:

$$\mathrm{loss}_{ce} = -log\left(\frac{\exp\left(\tilde{y}_{y(x)}(x)\right)}{\sum_c^C exp(\tilde{y}_c(x))}\right) \quad (9)$$

$$\mathrm{loss}_{dice} = 1 - \frac{2\sum_i y_i\tilde{y}_i}{\sum_i y_i + \sum_i \tilde{y}_i} \quad (10)$$

where x is a pixel in image domain $\Omega, \tilde{y}_c\colon \Omega \rightarrow \mathcal{R}$ is the predicted score for $c \in \{0, \dots, C\}$, C is the number of classes, and $y\colon \Omega \rightarrow \{0, \dots, C\}$ is the ground-truth segmentation map. Therefore, $\tilde{y}_{y(x)}(x)$ is the predicted score for ground-truth class $y(x)$ at image $\mathrm{x}(\mathrm{Eq.}\,(9))$. In Eq. (10), y_i is the ith output of the 1×1 convolution layer passed through a softmax function and $\tilde{y}_i$ is the ground-truth class.

The total loss is:

$$L = \theta\mathrm{loss}_{ce} + (1-\theta)\mathrm{loss}_{dice} \quad (11)$$

where $\theta \in [0, 1]$ controls the proportion of cross-entropy loss and dice loss to the total loss.

Transfer learning

To demonstrate the wider application of NEATmap for analyzing other cell marker labeled volumetric datasets beyond c-Fos immunofluorescence dataset, we employ transfer learning for

fine-tuning of our pre-trained 3D-HSFormer with iDISCO processed c-Fos dataset and FISH labeling of cell-type marker genes' datasets in thick brain slices.

Open source iDISCO whole mouse brain c-Fos dataset annotated by our training data preparation process (Supplementary Fig. 2) was used for fine-tuning of our pre-trained 3D-HSFormer. The iDISCO c-Fos mouse brain dataset was cut into 41 slices of $64 \times 1892 \times 1802$ pixels in size. Each slice was divided into 49 subvolumes of $64 \times 256 \times 256$ pixels in size, resulting in a total of 2009 subvolumes of 3D data for training (the training process was the same as described in the previous section). We randomly chose 10% of these subvolumes for validation. The test data is sourced from another iDISCO processed mouse brain c-Fos dataset, which has been annotated by an expert and validated by two other experts. Five slices of $64 \times 1778 \times 1802$ pixels in size were divided into 42 subvolumes of $64 \times 256 \times 256$ pixels in size for each of them.

For FISH labeled cell-type markers datasets, annotation was carried out by experts on a thick brain slice dataset of *Sst*, *Vglut1*, and *Vgat* genes respectively. The training dataset for each gene consists of one brain slice of $64 \times 3500 \times 2500$ pixels in size, divided into 70 subvolumes of $64 \times 256 \times 256$ pixels in size. The test data was prepared following the same protocol as training dataset on another brain slice. The training processes were following the same schedule as previous.

Automated whole brain dual-channel segmentation

The network designed in this process allows whole brain testing of c-Fos channel and autofluorescence channel in datasets of different animals. Each brain was sectioned into 50 slices approximately, and every brain slice was clipped into 70 subvolumes. We use the c-Fos imaging data of a mouse brain from FST group to train the 3D-HSFormer.

Evaluation metrics

We used three voxel-wise segmentation metrics, Dice, Sst and Jc to evaluate the segmentation accuracy of 3D-HSFormer. The Dice coefficient measures the similarity between the predicted segmentation map and the ground-true set of segmentation maps. The Dice coefficient is calculated as:

$$\text{Dice} = \frac{2|Y \cap \tilde{Y}|}{|Y| + |\tilde{Y}|} \tag{12}$$

where Y and $\tilde{Y}$ are the predicted segmentation set and the ground-true segmentation set, respectively. Dice $\in [0, 1]$, with 0 meaning no overlap and 1 meaning that the network predicts a perfect segmentation. The Dice value of ~0.7 indicates a good segmentation result, and Dice value of ~0.9 is close to ground-truth accuracy.

The Jaccard coefficient was used to compare the similarities and differences between the predicted set and the ground-truth set. The sensitivity describes the ratio of identified true positives to all true positives. The true positive (TP) indicates valid match, and false negative (FN) indicates that there is no valid match for the ground-truth segmentation. We use these values to calculate the sensitivity of the predicted segmentation results as:

$$\mathrm{Jc} = \frac{|Y \cap \tilde{Y}|}{|Y \cup \tilde{Y}|} \tag{13}$$

$$\mathrm{Sst} = \frac{TP}{TP+FN} \tag{14}$$

The range of values of Jc and Sst indicators is the same as the range of values of Dice indicator, and larger values indicate better results of prediction segmentation.

We use the Wilcoxon test to compare the performance of different algorithms, and each group of data is the predicted segmentation result of each algorithm applied to the same brain slice clipped into 70 subvolumes.

Post-processing

The predicted segmentation subvolumes of dual-channel images were obtained in the Test phase and were spliced according to their positions in the raw image coordinates (Fig. 1f). First, the Autofluorescence filter was utilized to clear the segmented results of the network predicted autofluorescence signal in the c-Fos channel. The Autofluorescence filter can be formulated as:

$$F = B - (B \cap A) \tag{15}$$

where set B and set A are the segmentation results of c-Fos channel and autofluorescence channel respectively by 3D-HSFormer. Set F is the result of 3D-HSFormer segmentation of c-Fos$^+$ signal after Autofluorescence filter. Secondly, the No-soma filter was used to remove unreasonably small or large objects from the network predicted segmentation results. Finally, the Intensity-based prediction filter was designed to profile the average signal intensities of detected c-Fos$^+$ cells across the whole brain at voxel level. Then the segmentation results can be selectively output based on the user specified intensity ranges. The Intensity-based prediction filter can be formulated as:

$$M(x_{\mathrm{seg}}, y_{\mathrm{seg}}, z_{\mathrm{seg}}) = \begin{cases} 0 & \text{if } i < \text{min intensity} \\ m & \text{if min intensity} \le i \le \text{max intensity} \\ 0 & \text{if } i > \text{max intensity} \end{cases} \tag{16}$$

$$i = I(x_{\mathrm{seg}}, y_{\mathrm{seg}}, z_{\mathrm{seg}}) \tag{17}$$

where $M(\cdot)$ is the image under c-Fos channel, x_{seg}, y_{seg} and z_{seg} are the coordinates of the filtered segmentation mask result, and m is the c-Fos$^+$ mask under the corresponding coordinates. $I(\cdot)$ is the image under c-Fos channel, and i is the c-Fos$^+$ signal intensity under corresponding coordinates.

Brain registration and cell counting

For brain registration, we used the average template, annotation file of the Allen mouse brain atlas (CCFv3). The reconstructed brain was registered with a reference autofluorescence brain template with

Elastix [14] (http://elastix.lumc.nl). The primary process of brain registration involves following steps: firstly, down-sampling the whole brain 3D images into $25 \times 25 \times 25\, \mu m^3$ voxel sized; secondly, employing Adaptive Stochastic Gradient Descent (ASGD) as the optimizer for non-rigid B-spline transformation; thirdly, utilizing Advanced Mattes Mutual Information as the similarity metric; and finally, employing three image pyramids with variable resolutions for interpolation and resampling. Transformation parameters of brain alignment were used to obtain the coordinates of cells in the whole brain. The original 3D volume of the predicted segmentation result was down-sampled to $25 \times 25 \times 25\, \mu m^3$ voxel size, then flattened and sutured to reconstruct the entire brain. The segmented images output in the *Post-processing* phase are labeled and analyzed in the connectivity domain (connectivity = 4) to obtain the centroid coordinates and volume of each c-Fos$^+$ cells. For each c-Fos$^+$ cell, the physical coordinate was calculated as centroid coordinates (x, y, z) multiple with voxel size $(4\, \mu m)$. The cell counting was completed with previously reported tool Freesia (https://github.com/BilabOptics/freesia-mapping) in each brain area of the whole brain.

Quantification and statistics

We performed whole brain inference of data of animals in two behavioral tests (a total of 30 mice) with the already trained neural network. The cell density was quantified number of c-Fos$^+$ neurons per volume μm^3 and the volume ratio was computed as fraction of total volume of c-Fos$^+$ neurons in the region over the total region volume. The physical size of c-Fos$^+$ neurons was set to 24 μm according to the average measurement size (Supplementary Fig. 3e, f).

The Wilcoxon signed-rank test was utilized to assess significant differences in the performance of various metrics among deep learning models. For determining significant differences in the number of c-Fos$^+$ neurons between groups, we employed the student's t-test. To control the false detection rate for multiple comparisons, the false discovery rate (FDR) q-value was calculated, with sample size (n), as shown in the main text and supplementary figure legends. The statistical tests mentioned above are implemented using the SciPy library https://scipy.org/ in Python. The Benjamini-Hochberg multiple comparison test [15] was used to control for the FDR. Cutoff fold change > 1.4 or < -1.4 and adjusted p-values < 0.01 were employed to select regions with significant differential expression of c-Fos$^+$ neurons between the SDS group and the ES group.

The relative z-score indicates how much the specific value of an individual animal either in the experimental group or in the Ctrl group differs from the mean of the Ctrl group over the standard deviation of the Ctrl group. More precisely, for each animal, the relative z-score was calculated by following equation

$$z_i = \frac{r_i - \bar{r}_{ctrl}}{sd_{ctrl}} \quad (18)$$

where r_i is the number of c-Fos$^+$ neurons in the particular brain area of ith animal either from the experimental group or Ctrl group, $\bar{r}_{ctrl}$ is the mean number of c-Fos$^+$ neurons of all Ctrl animals in that brain area and sd_{ctrl} is the standard deviation of the number of c-Fos$^+$ neurons of all Ctrl animals in that brain area (Fig. 6a).

The coefficient of variation (CV) is the ratio of the standard deviation to the mean and shows the extend of variability over the mean of the group. In the Fig. 6b, the coefficient of variation in the SDS group and the ES group were calculated as following:

$$cv_g = \frac{sd_g}{mean_g} \tag{19}$$

where cv_g represents the CV for either the SDS group or the ES group, sd_g and $mean_g$ correspond to the standard deviation and mean of the number of c-Fos$^+$ neurons in the respective group.

We utilize Spearman correlation to assess the correlation between brain areas in the Fig. 6e, f. The formula is as following:

$$\rho = 1 - \frac{6\sum_{i=1}^{n} d_i^2}{n(n^2-1)} \tag{20}$$

where $\rho \in [-1,1]$ is Spearman correlation coefficient, and n represents the total number of animals in the group. In the Fig. 6e, d_i represents the difference between the two ranks (from low to high) of c-Fos$^+$ neurons in different brain areas for the ith animal. Similarly, d_i represents the rank difference in c-Fos$^+$ neuron numbers in the same brain area for the ith animal between the SDS group and the ES group (Fig. 6f).

Data availability

The openly available c-Fos whole-mount immunostaining data obtained with iDISCO can be found at (https://osf.io/5qzn7/). The total size of the mouse whole-brain dual-channel VISoR imaging datasets (both raw and processed) of this study exceeded 10 Terabytes. Uploading it to a public data repository is impractical. We prepared an instance dataset of a mouse whole-brain dual-channel imaging for validating the automated segmentation results of NEATmap, including Fig. 1a, Supplementary Videos 1 and 2 of dual-channel brain slices and segmentation results, as well as high-resolution mouse brain slices showing c-Fos$^+$ cells in Fig. 1e (available at https://zenodo.org/record/8133486).

Code availability

The source code for all modules of NEATmap is implemented in Python and has been deposited in a public online repository (https://github.com/mesobrain/NEATmap_code). Software for NEATmap can be found in the NEATmap GitHub repository: https://github.com/mesobrain/NEATmap_software.

References

1. Xu, F., et al. *High-throughput mapping of a whole rhesus monkey brain at micrometer resolution.* Nature Biotechnology, 2021. 39(12): p. 1521-1528.
2. Wang, H., et al. *Scalable volumetric imaging for ultrahigh-speed brain mapping at synaptic resolution.* National Science Review, 2019. **6**(5): p. 982-992.
3. Marstal, K., et al. *SimpleElastix: A User-Friendly, Multi-lingual Library for Medical Image Registration*. in *2016 IEEE Conference on Computer Vision and Pattern Recognition Workshops (CVPRW)*. 2016.
4. Schaefer, S., T. McPhail, and J. Warren, *Image deformation using moving least squares.* ACM Trans. Graph., 2006. **25**(3): p. 533–540.
5. Chen, J., et al. *The Allen Cell and Structure Segmenter: a new open source toolkit for segmenting 3D intracellular structures in fluorescence microscopy images.* bioRxiv, 2020: p. 491035.
6. Canny, J. *A Computational Approach to Edge Detection.* IEEE Transactions on Pattern Analysis and Machine Intelligence, 1986. **PAMI-8**(6): p. 679-698.
7. Dosovitskiy, A., et al. *An Image is Worth 16x16 Words: Transformers for Image Recognition at S cale*. in *International Conference on Learning Representations*. 2021.
8. Yu, F. and V. Koltun. *Multi-Scale Context Aggregation by Dilated Convolutions*. in *International Conference on Learning Representations*. 2016.
9. Liu, Z., et al. *Swin Transformer: Hierarchical Vision Transformer using Shifted Windows*. in *2021 IEEE/CVF International Conference on Computer Vision (ICCV)*. 2021.
10. Cao, H., et al. *Swin-Unet: Unet-Like Pure Transformer for Medical Image Segmentation*. in *Computer Vision – ECCV 2022 Workshops*. 2023. Cham: Springer Nature Switzerland.
11. Ronneberger, O., P. Fischer, and T. Brox. *U-Net: Convolutional Networks for Biomedical Image Segmentation*. in *Medical Image Computing and Computer-Assisted Intervention – MICCAI 2015*. 2015. Cham: Springer International Publishing.
12. Paszke, A., et al. *Pytorch: An imperative style, high-performance deep learning library.* Advances in neural information processing systems, 2019. **32**.
13. Drozdzal, M., et al. *The importance of skip connections in biomedical image segmentation*. in *International Workshop on Deep Learning in Medical Image Analysis, International Workshop on Large-Scale Annotation of Biomedical Data and Expert Label Synthesis*. 2016. Springer.
14. Klein, S., et al. *Elastix: a toolbox for intensity-based medical image registration.* IEEE transactions on medical imaging, 2009. **29**(1): p. 196-205.
15. Benjamini Y and Hochberg Y. Controlling the false discovery rate: A practical and powerful approach to multiple testing. J. R. Stat. Soc. Ser. B Methodol. 1995; **57**: 289–300.

349 # Supplementary figure

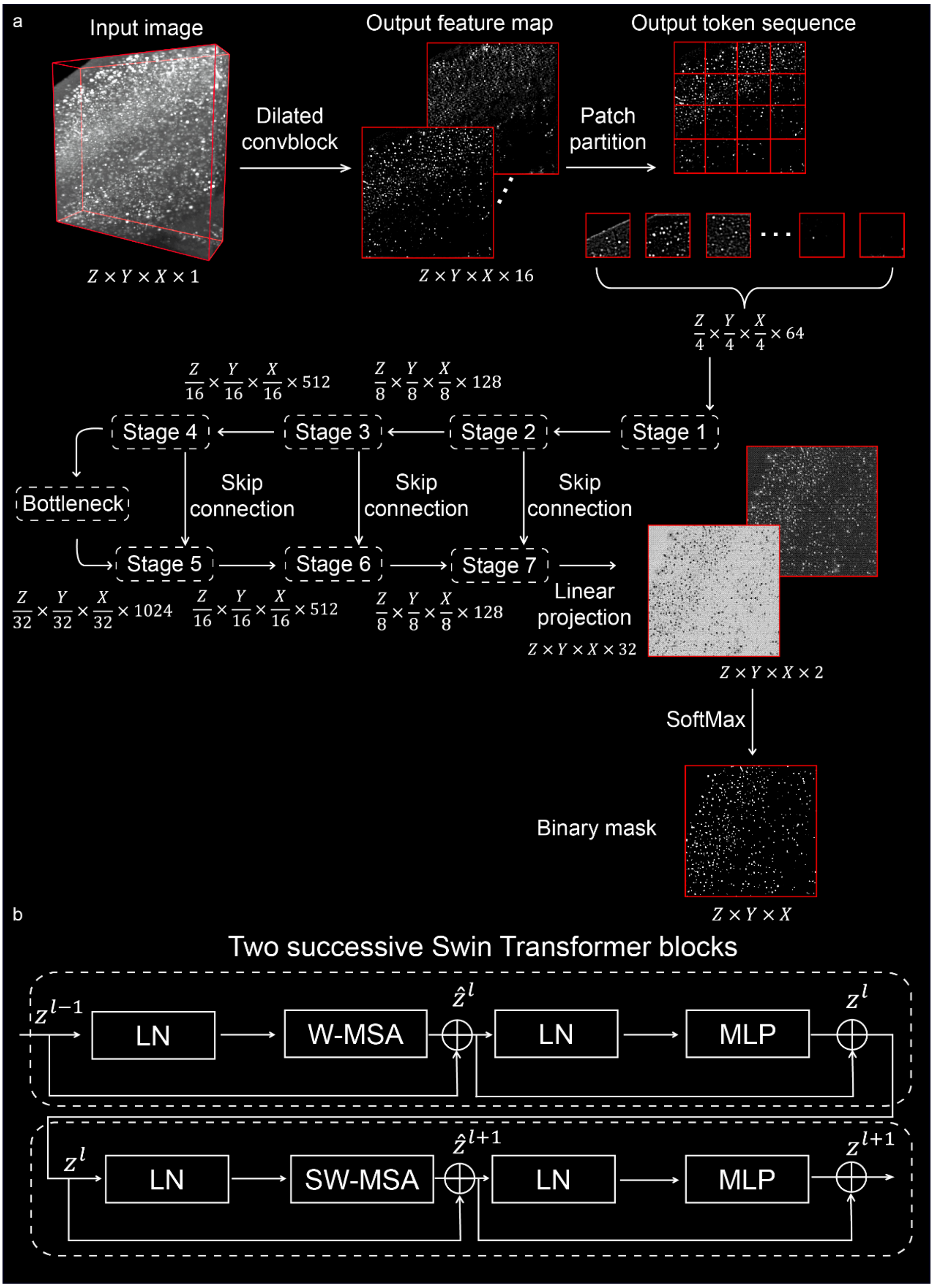

Supplementary Fig. 1 Details of the 3D-HSFormer architecture implementation for segmentation. **a**, Details of the 3D-HSFormer segmentation, in conjunction with Figure 1. **b**, The composition of the two successive Swin Transformer blocks.

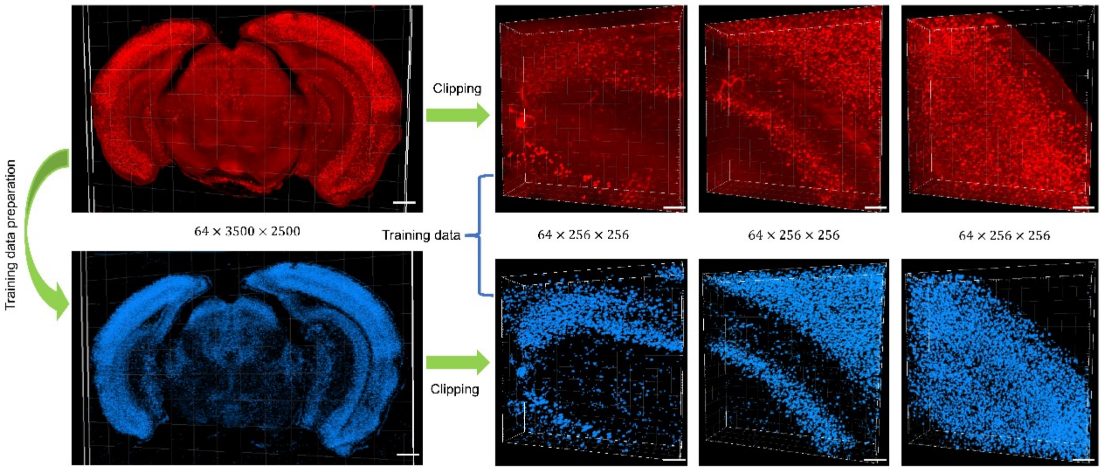

Supplementary Fig. 2 Training datasets preparation. Preprocessing flowchart of training data for artificial neural network. The red row represents the clipping process of the c-Fos channel images of an entire brain slice. The blue row represents the clipping process of the training data preparation results. The $z \times x \times y$ size of the brain slice (Scale bars, 1000 μm) is 64×3500×2500, and $z \times x \times y$ size of the clipping result (Scale bars, 150 μm) is $64 \times 256 \times 256$.

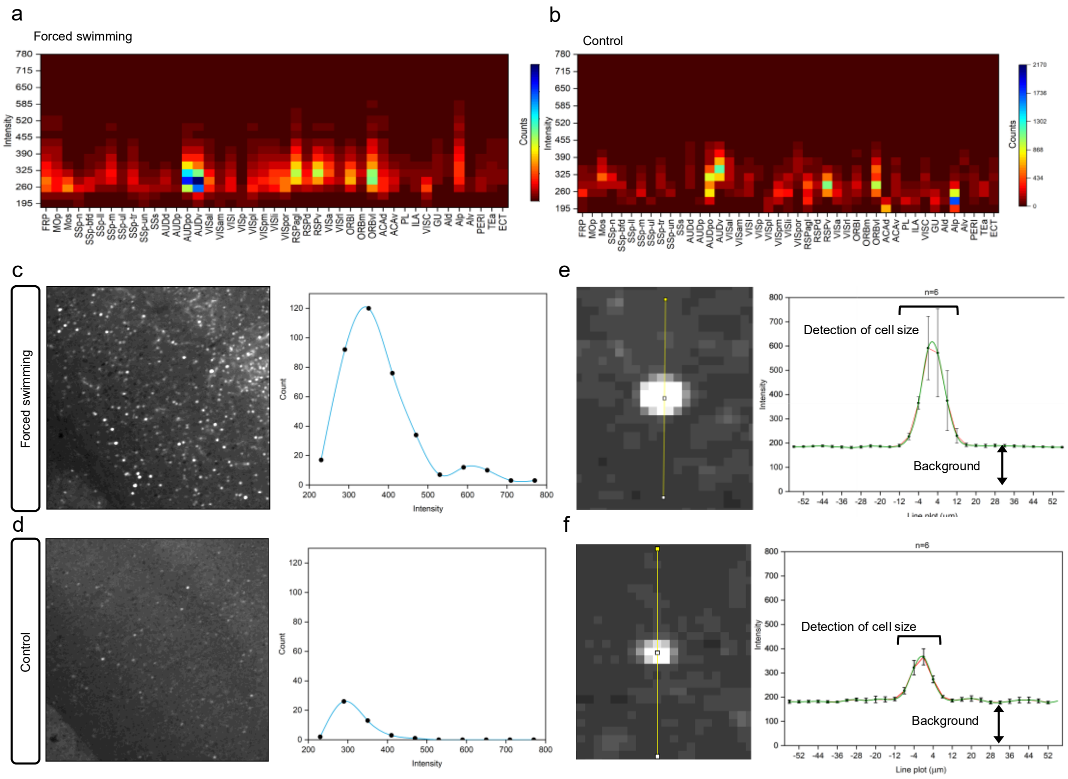

Supplementary Fig. 3 c-Fos⁺ immunofluorescence signal intensity analysis. **a**, **b**, The segmented putative cells in the Isocortex of FST group (left) and Ctrl group (right) are counted and plotted by the maximum intensity in heat maps. **c**, **d**, Profiling of c-Fos⁺ cells distribution by immunofluorescence signal intensity. Left column is a patch of raw instance images in the FST group and Ctrl group. The right column shows intensity distribution of the detected cells. The number of cells is plotted as black dots and fitted with B-spline curve. **e**, **f**, The size of the detected cells is measured by averaging multiple cells. The intensity profile of a typical weak c-Fos⁺ cell is around 350, for a background of 200, while a highly activated cell has a peak intensity typically above 600 (n = 6). The diameter of detected c-Fos cells is around 20 μm.

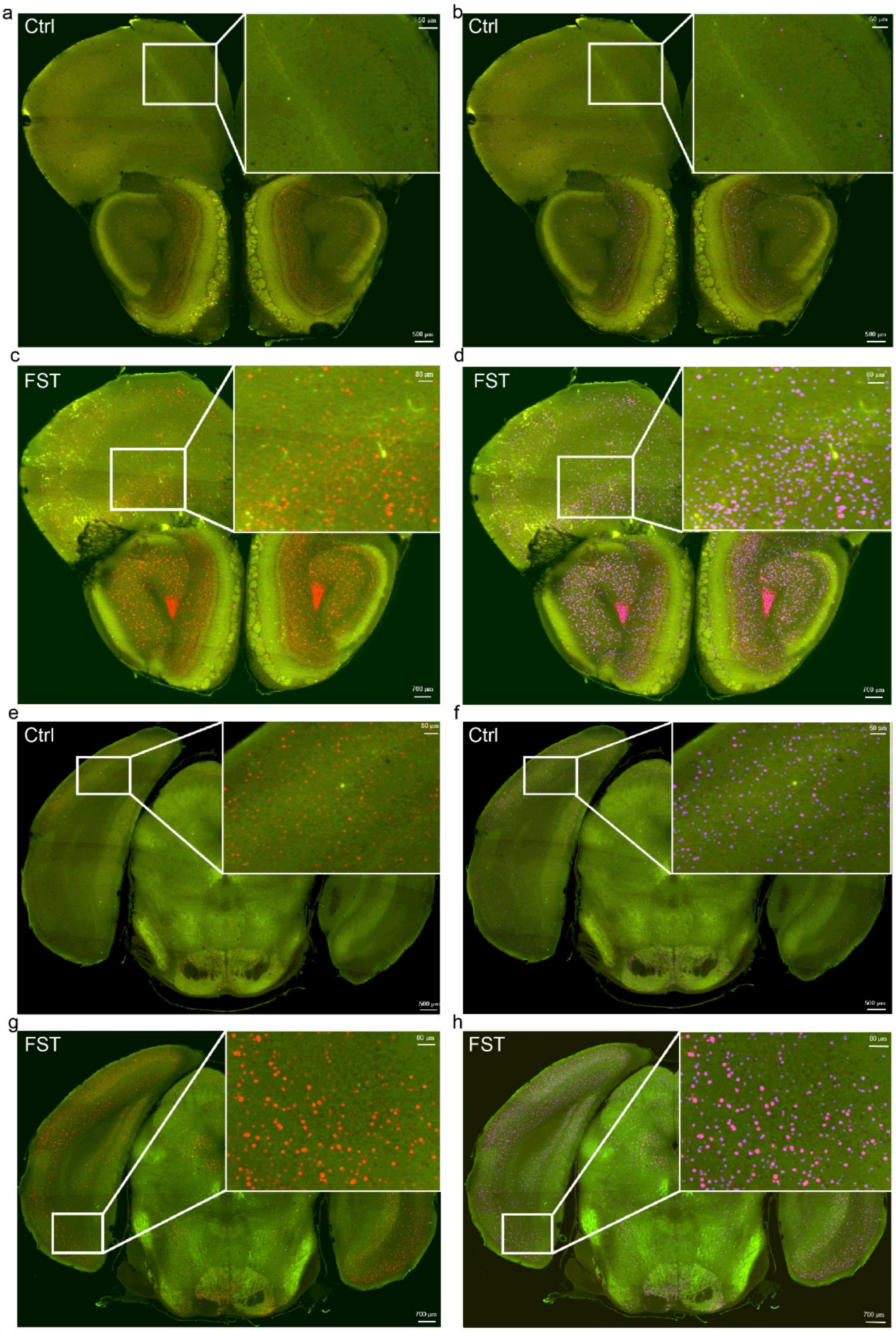

Supplementary Fig. 4 The segmentation results of c-Fos$^+$ cells in the anterior and posterior parts of brains are compared between the FST group and the Ctrl group. Red: c-Fos channel, green: autofluorescence channel, blue: segmented c-Fos$^+$ cells.

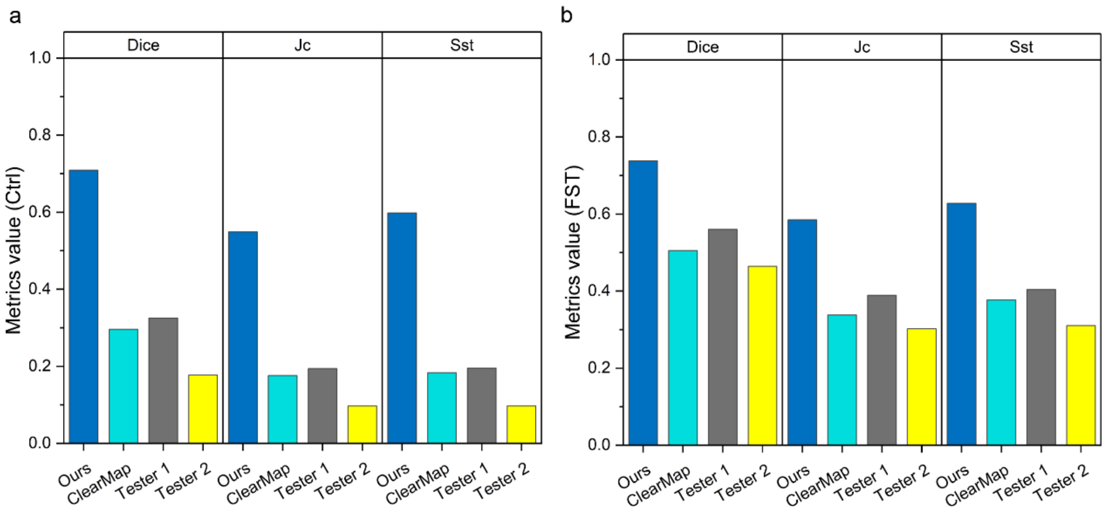

Supplementary Fig. 5 Comparison of the segmentation performance of NEATmap and other methods by evaluation metrics Dice, Jc and Sst.

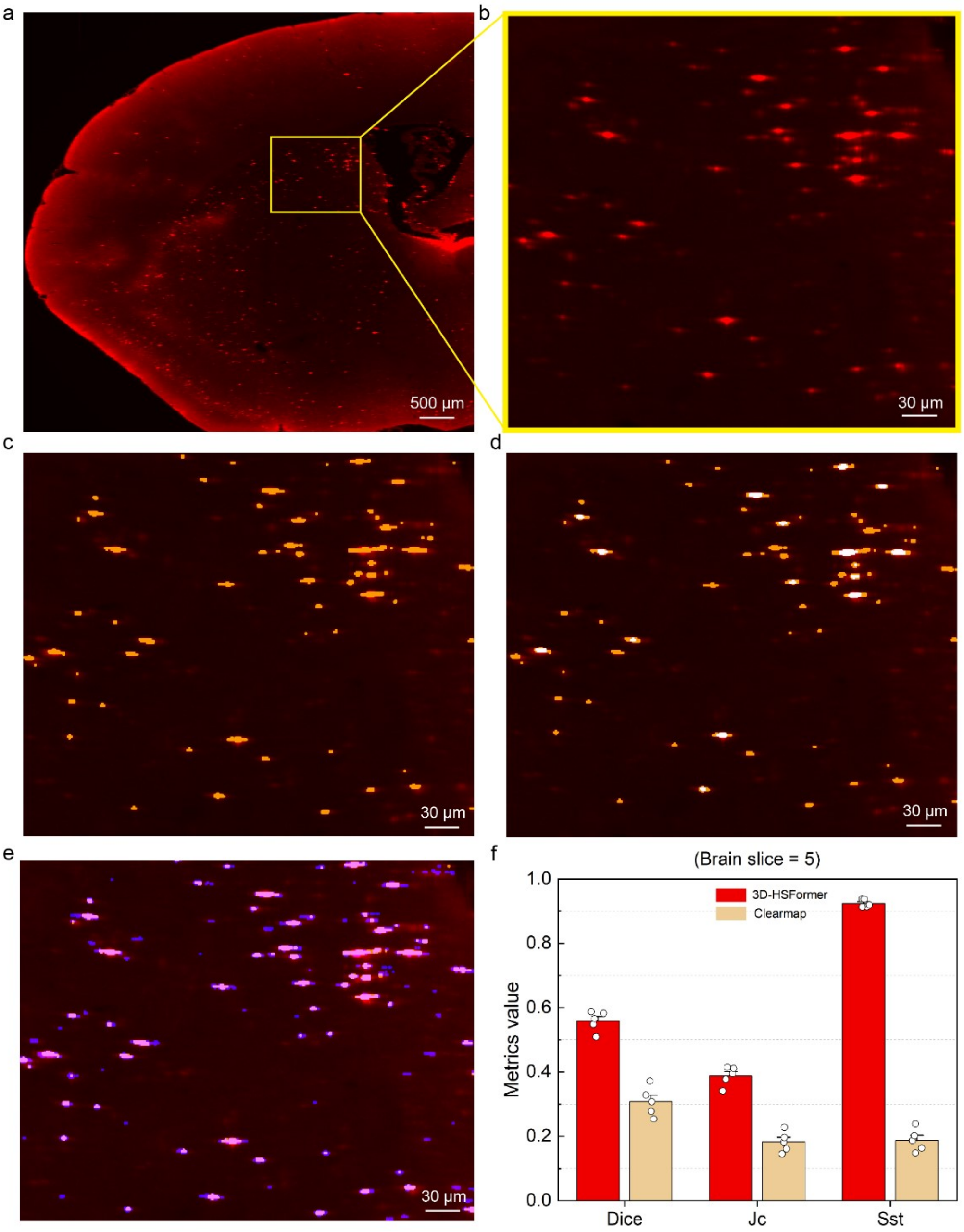

Supplementary Fig. 6 Performance of 3D-HSFormer on iDISCO cleared whole-brain images. **a**-**e**, Expression of c-Fos signal in the brain under iDISCO clearing, along with the presentation of ground truth, ClearMap, and 3D-HSFormer segmentation results. Red: c-Fos channel, brownness: ground truth, sky blue: ClearMap segmentation c-Fos^{+} results, blue: 3D-HSFormer segmentation c-Fos^{+} results. The combination of sky blue and brown creates the appearance of white. Likewise, when blue is combined with brown, the result appears pink. **f**, Comparison of segmentation accuracy between 3D-HSFormer and ClearMap on 5 brain sections (thickness 192 μm per).

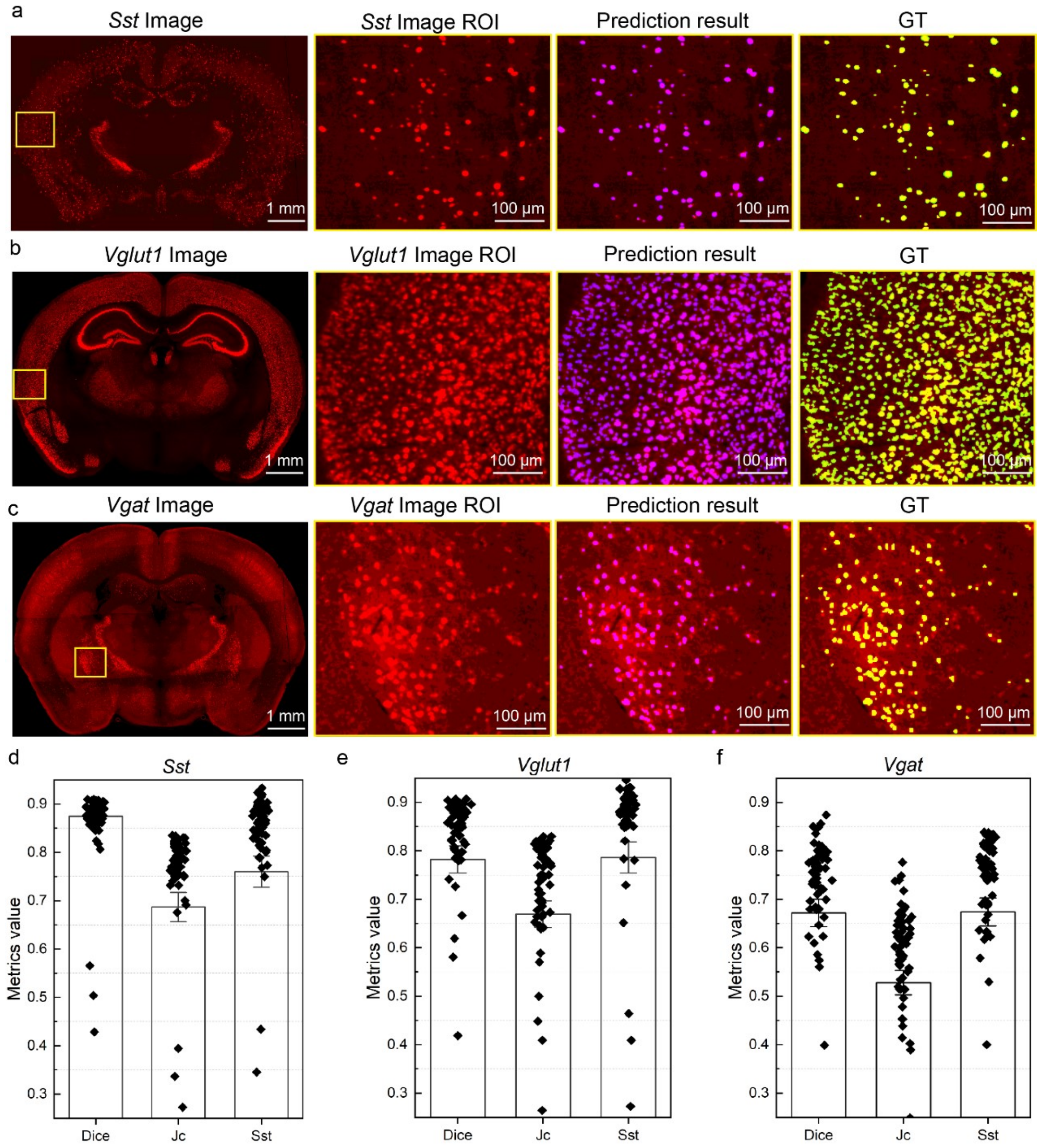

Supplementary Fig. 7 Application of 3D-HSFormer on cell segmentation of FISH labeled *Sst*, *Vglut1* and *Vgat* genes. **a-c**, Images of *Sst* (**a**), *Vglut1* (**b**) and *Vgat* (**c**) expression cells in thick brain slices, along with 3D-HSFormer prediction results and ground truth. Red: gene expression signal, blue: segmentation results, green: ground truth (GT). The combination of blue and red creates the appearance of magenta. Likewise, when green is combined with red, the color appears as yellow. **d**-**f**, Evaluation metrics of voxel-wise segmentation of cells by 3D-HSFormer on *Sst* (**d**), *Vglut1* (**e**) and *Vgat* (**f**) FISH labeling datasets. The number of subvolumes used for test is 70 for each gene.

351

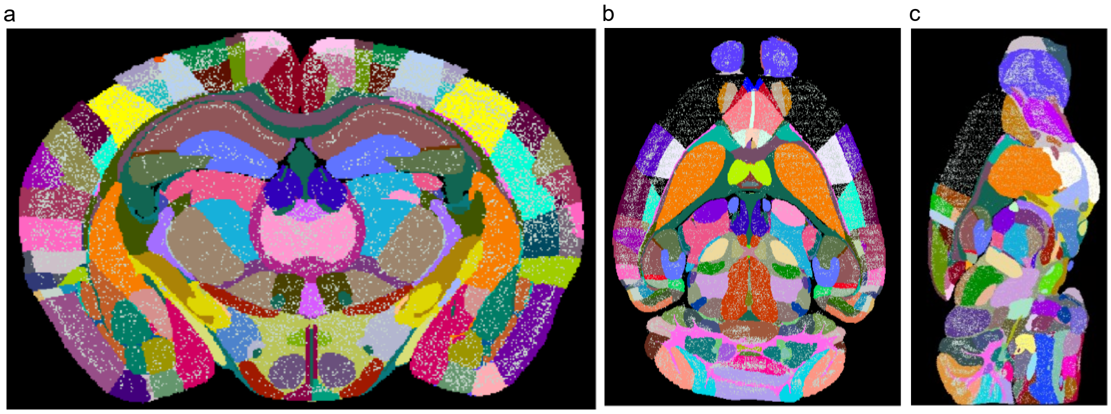

Supplementary Fig. 8 Segmentation results are mapped to the ABA CCFv3. a-c, Whole brain segmentation results of c-Fos$^+$ cells are overlaid on the randomly color-coded atlas to reveal different brain areas, showing at coronal plane (**a**), horizontal plane (**b**) and sagittal plane (**c**).

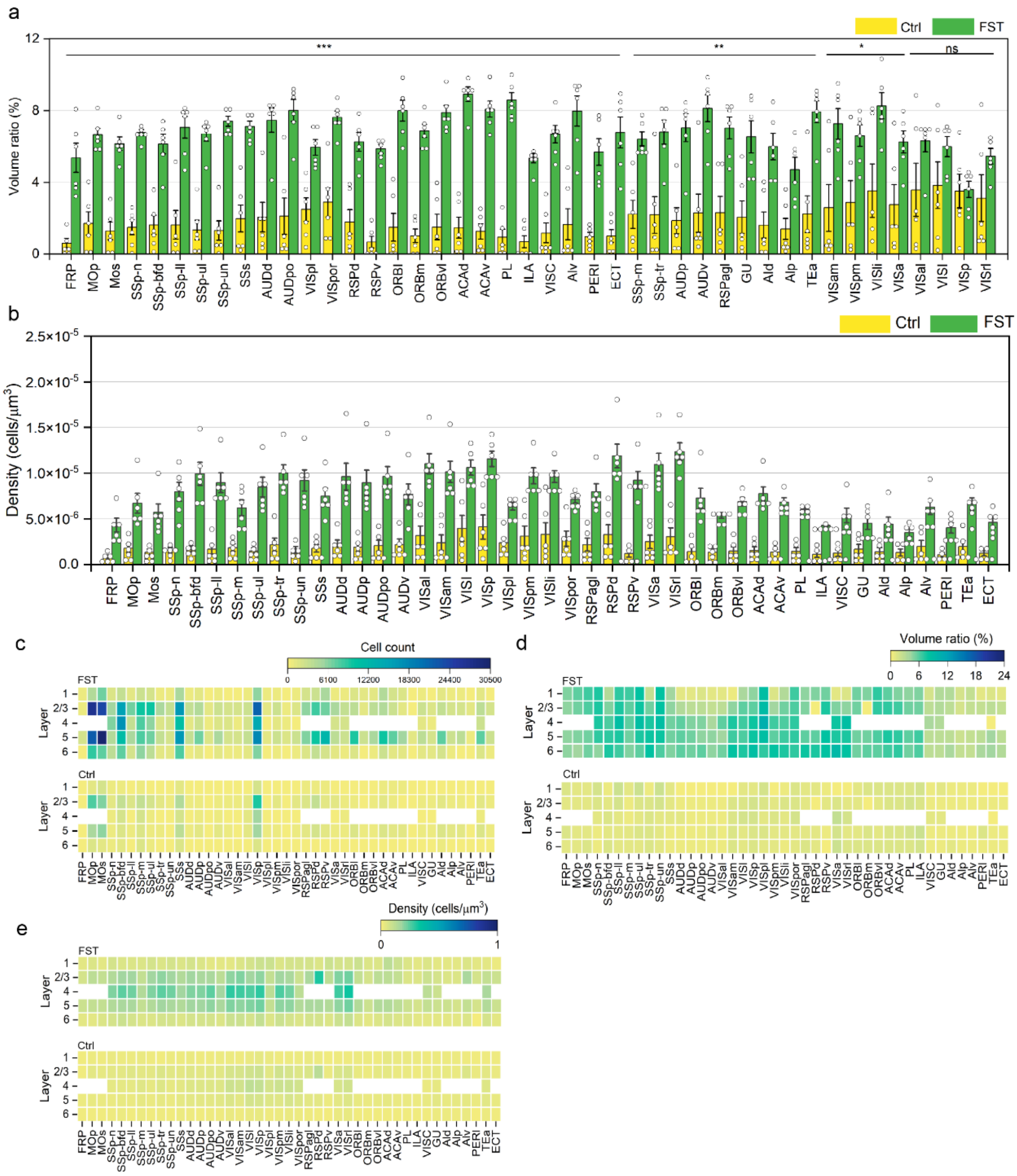

Supplementary Fig. 9 Quantitative analysis of whole brain neuronal activation by cell count, volume ratio and cell density in FST and Ctrl mice. **a**, **b**, The volume ratio and density of c-Fos$^+$ cells in the Isocortex. Data is shown mean ± SE (n = 6). **c**, Layer-wise c-Fos$^+$ cell in Isocortex of FST group and Ctrl group (n = 12). **d**, Layer-wise c-Fos$^+$ volume ratio in Isocortex of FST and Ctrl groups (n = 12). **e**, Layer-wise c-Fos$^+$ density in Isocortex of FST and Ctrl groups (n = 12).

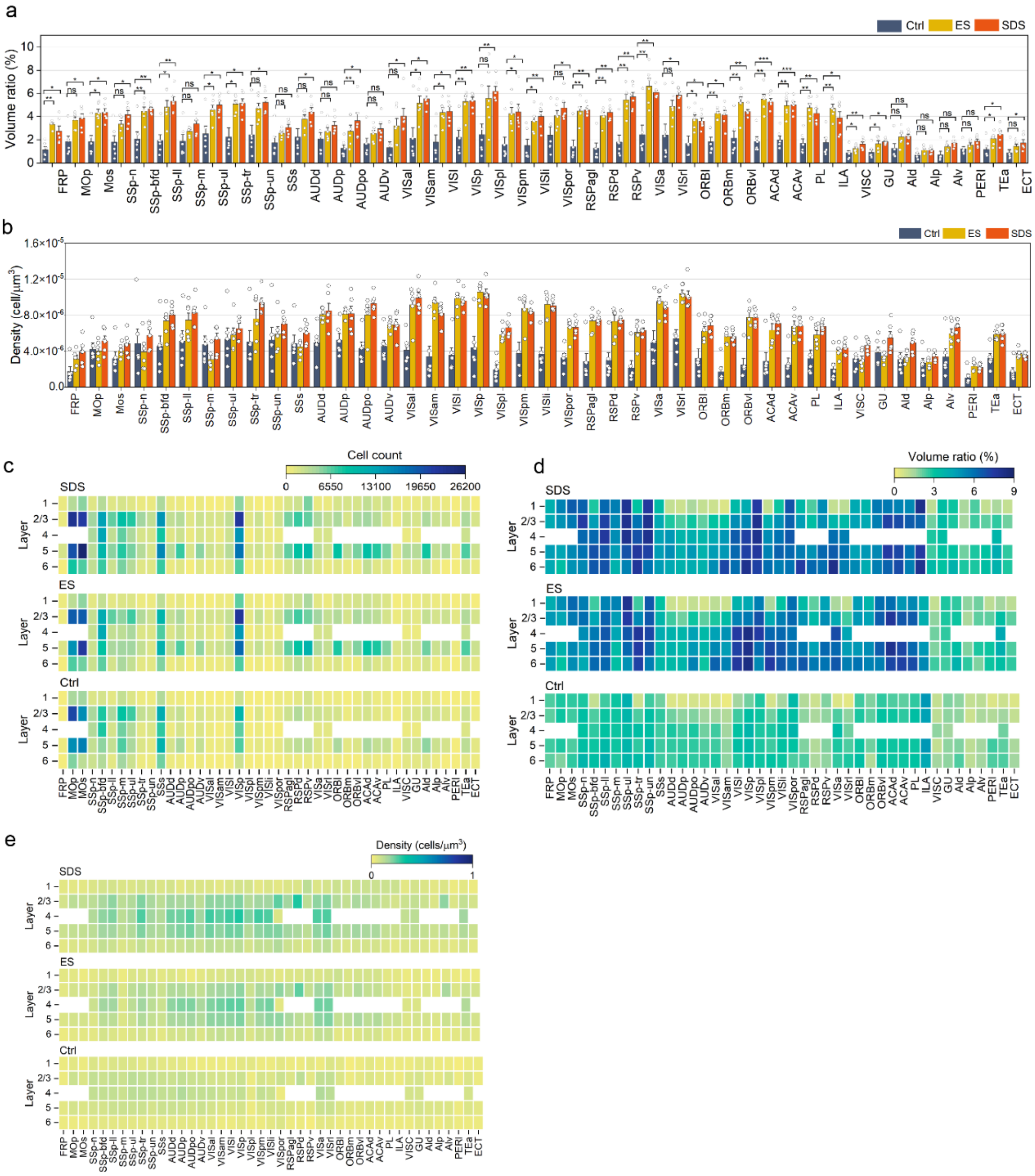

Supplementary Fig. 10 Quantitative analysis of whole brain neuronal activation by cell count, volume ratio and cell density analysis in animals had acute social stress and emotional stress test. a, **b**, The volume ratio and cell density of c-Fos$^+$ cells in the Isocortex. Data is shown as mean ± SE (n = 6). **c**, Layer-wise c-Fos$^+$ cell count in Isocortex of SDS, ES and Ctrl groups (n = 18). **d**, Layer-wise c-Fos$^+$ volume ratio in Isocortex of SDS, ES and Ctrl groups (n = 18). **e**, Layer-wise c-Fos$^+$ cell density in Isocortex of SDS, ES and Ctrl groups (n = 18).

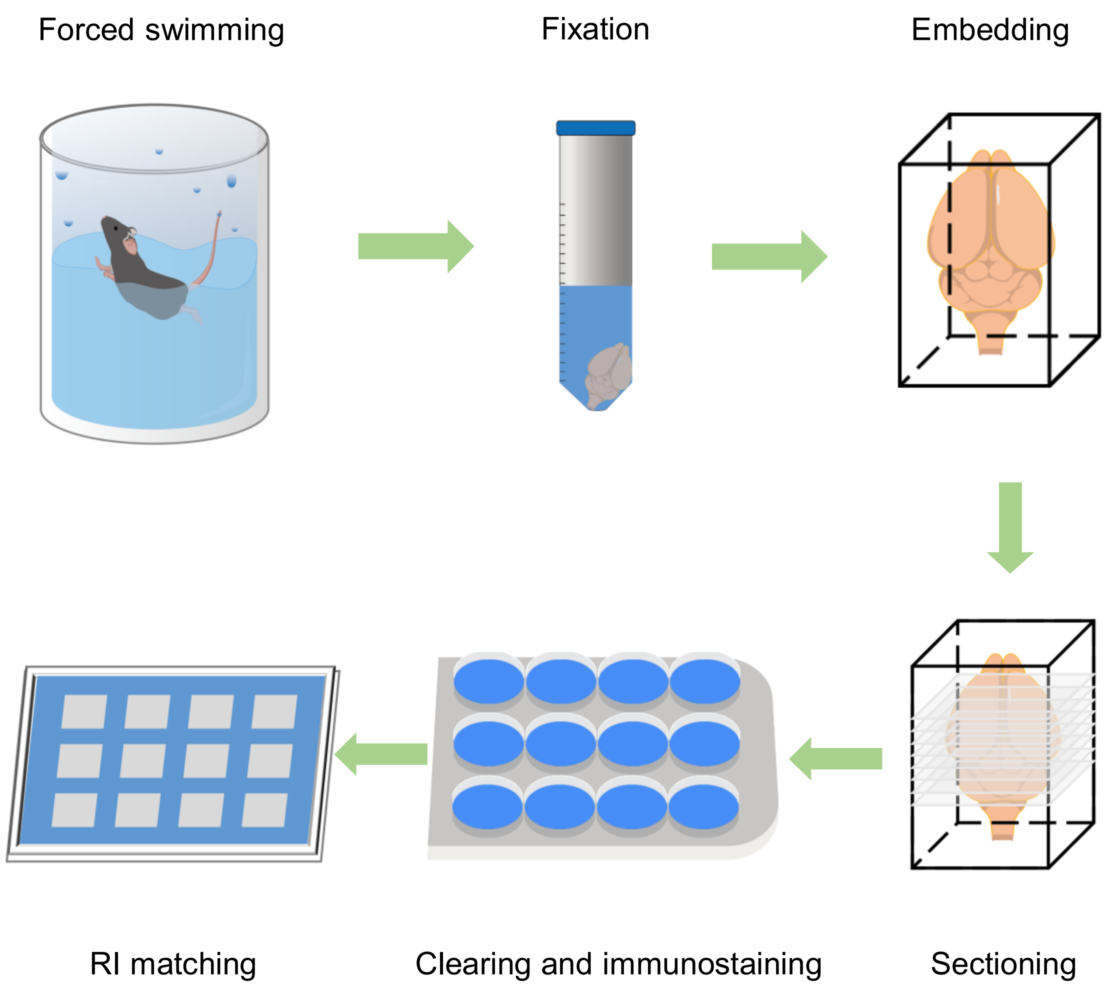

Supplementary Fig. 11 Sample preparation protocol whole brain c-Fos imaging. The sample preparation process has the following key steps: brain harvesting, embedding, sectioning, tissue clearing, immunostaining, mounting and refractive index matching.

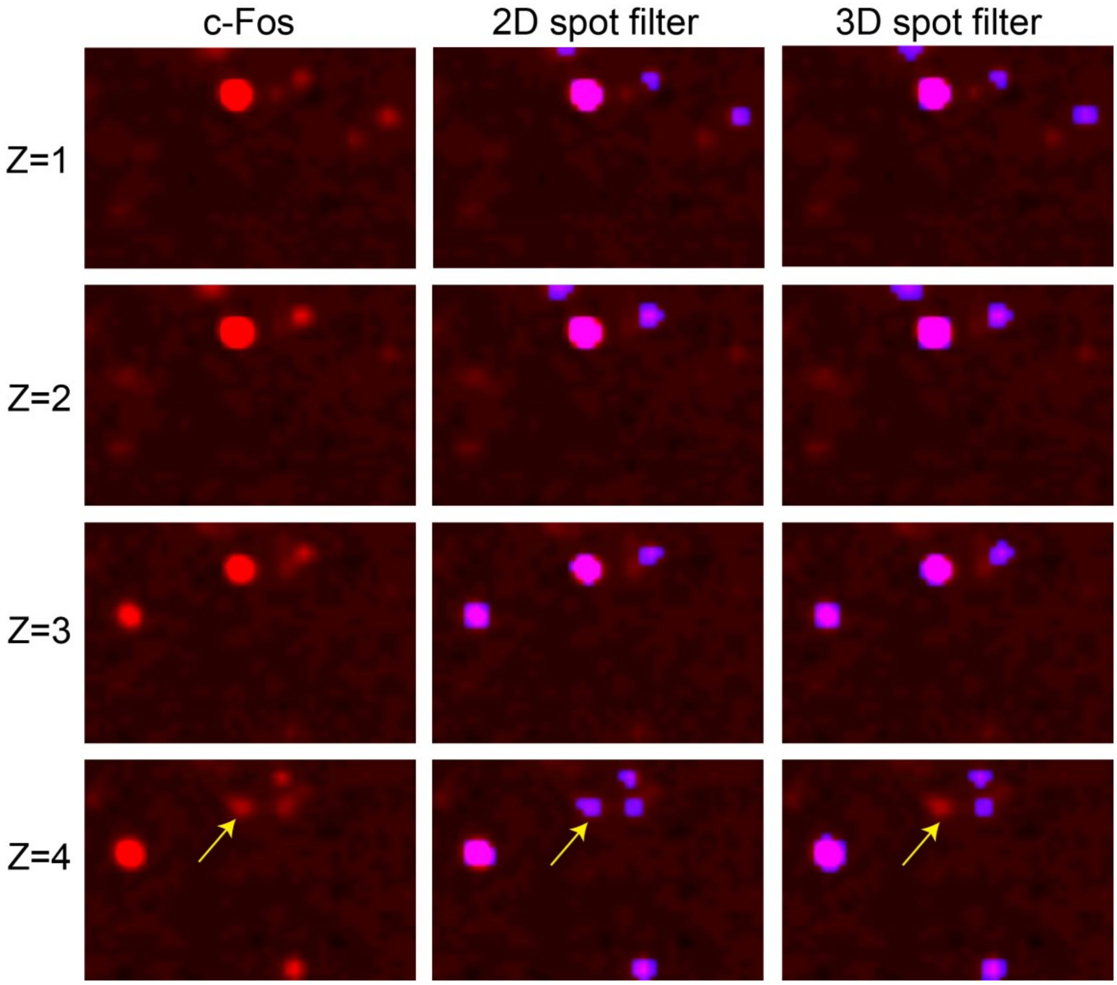

Supplementary Fig. 12 Comparison between 2D and 3D spot filters applied to c-Fos image stack. Four successive z-slices are presented in four rows. The first column displays the c-Fos signal channel in red. The second and third columns show the results of 2D spot filter and 3D spot filter detection over the original c-Fos signal. Blue indicates the mask results, while overlapping areas are presented in magenta.

352

Supplementary video

Supplementary Video 1. Example of whole brain c-Fos immunolabeling and reconstructed volumetric image data. Visualization of the whole brain as well as the brain activity map of the brain division layers with the voxel size of $4 \times 4 \times 4\ \mu m^3$. The 3D-HSFormer whole brain segmentation and the segmentation details in the cortex region are presented.

Supplementary Video 2. Comparative visualization of segmentation results in cortical regions for the FST and control groups. A large number of neurons were activated in the cortex of the brain in the FST group.

Supplementary Video 3. Visualization of whole brain segmentation results mapped to the Allen Brain Atlas. For clarity of visualization, we down-sampled the size of the whole brain segmentation result voxels to $25 \times 25 \times 25\ \mu m^3$.

Supplementary Video 4. Heat map visualization comparison of c-Fos cell count in the FST and control groups.

Supplementary Video 5. The segmentation results are presented as a heat map of whole brain c-Fos activity and the distribution of expression in different brain areas. The video also shows the distribution of segmented c-Fos objectives in layered areas in brain areas of the cortex.

Supplementary Video 6. Experimental filming record of the forced swimming test. In general, mice exhibit three behaviors: Climbing, Swimming and Immobility. The total duration of the experiment was 5 minutes, the first 1 minute was for the mice to adapt to the temperature and environment of the water, and the last 4 minutes were devoted to analyzing the mice's behavior. Climbing refers to the behavior of mice climbing upwards along the inner wall of the beaker using their front paws. Swimming refers to the mice swimming around on a horizontal surface. Immobility was defined as behavior other than active movement upward avoiding submersion in water. The video records the second 4 minutes of the experiment with a total of 6 mice.

Supplementary Video 7. Example of raw c-Fos neuronal activity map for volumetric light sheet imaging with voxels of $1 \times 1 \times 3.5\ \mu m^3$. The high level of FST stimulation resulted in the activation of a large number of neurons in the mouse brain.

Supplementary table

Supplementary Table 1. Time consumption of each phase of the method.

Supplementary Table 2. The time consumption of various methods for achieving whole-brain segmentation.

Supplementary Table 3. The number and expression levels of c-Fos expressing cells in brains after forced swimming test. The sheet lists the number of c-Fos^{+} cells in all brain areas defined in the ABA CCFv3 for forced swimming group and control group.

Supplementary Table 4. The number of c-Fos-expressing cells in each subregion of the Isocortex. The first twelve tables list the number of cells expressed in different individuals and the thirteenth table lists the FDR q-values used for statistical hypothesis testing compared to the Ctrl group.

Supplementary Table 5. The number of cells expressed by c-Fos in each brain area of level 6 in the ABA CCFv3. A list the names of brain areas, B-M lists the number of cells expressed by c-Fos in the 12 mice, and N-P lists the p-values, FDR q-values, and corresponding significance levels.

Supplementary Table 6. Statistical hypothesis testing results of the experimental group and control group in bilateral brain areas (level 6). Columns B and C represent the FDR q-values for the left brain and right brain, respectively. Columns D and E indicate the corresponding significance levels. The G-K column represents brain areas where there is no significant difference in the left hemisphere and significant differences in the right hemisphere. Conversely, the M-Q column represents brain areas where there is no significant difference in the right hemisphere and significant differences in the left hemisphere.

Supplementary Table 7. The number of cells expressed by c-Fos in each brain area of level 6 in the ABA CCFv3 after social defeat emotional stress test. A list the names of brain areas, B-S lists the number of cells expressed by c-Fos in the 18 mice, and T-U lists the FDR q-values.

Supplementary Table 8. The relative z-score and coefficient of variation of the whole brain.
