# Supplementary figures and images for: NEATmap: a high-efficiency deep learning approach for whole mouse brain neuronal activity trace mapping
Source: Natl Sci Rev. 2024 Mar 26;11(5):nwae109. doi: 10.1093/nsr/nwae109 (PMC11145917; doi:10.1093/nsr/nwae109)

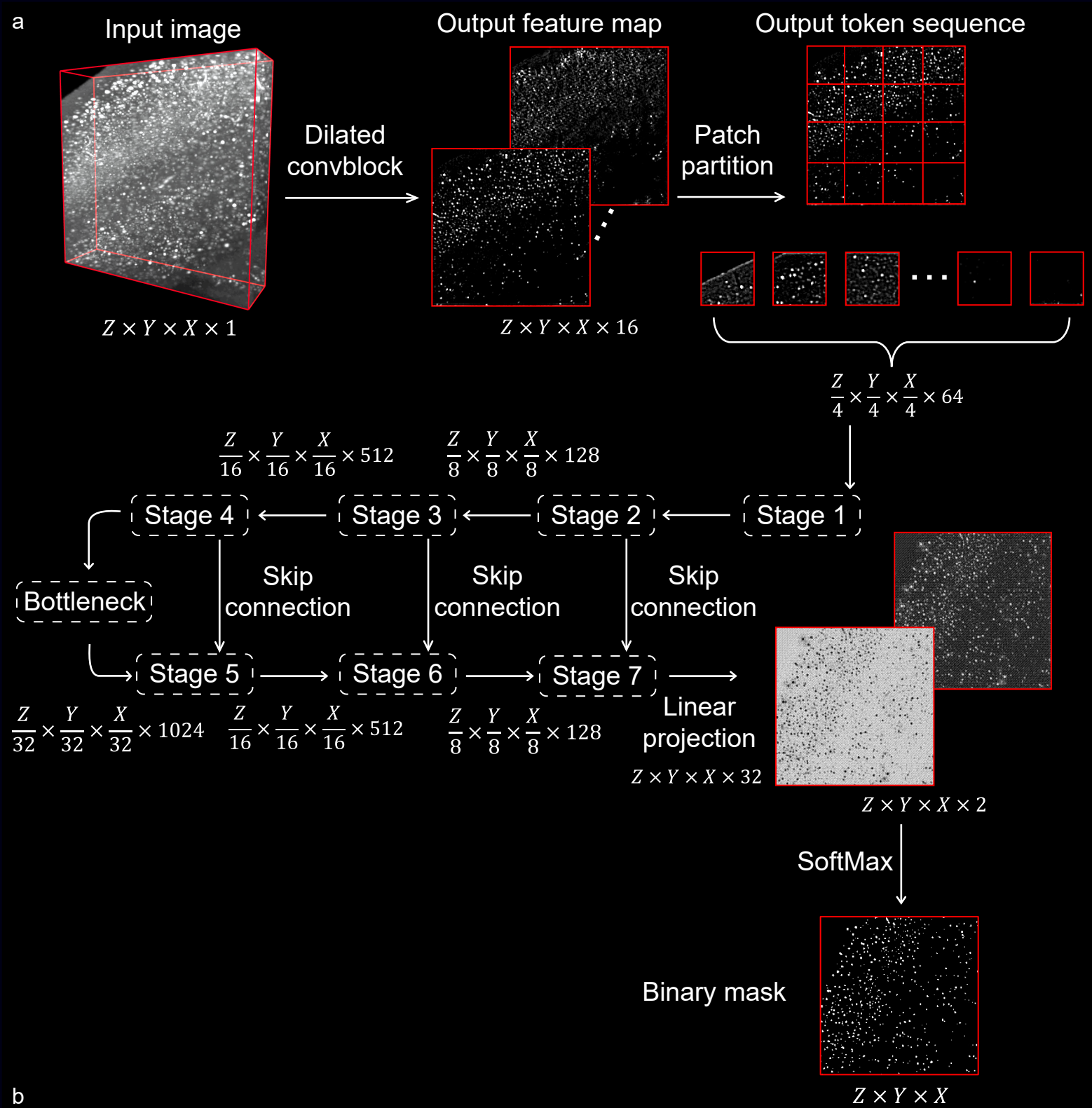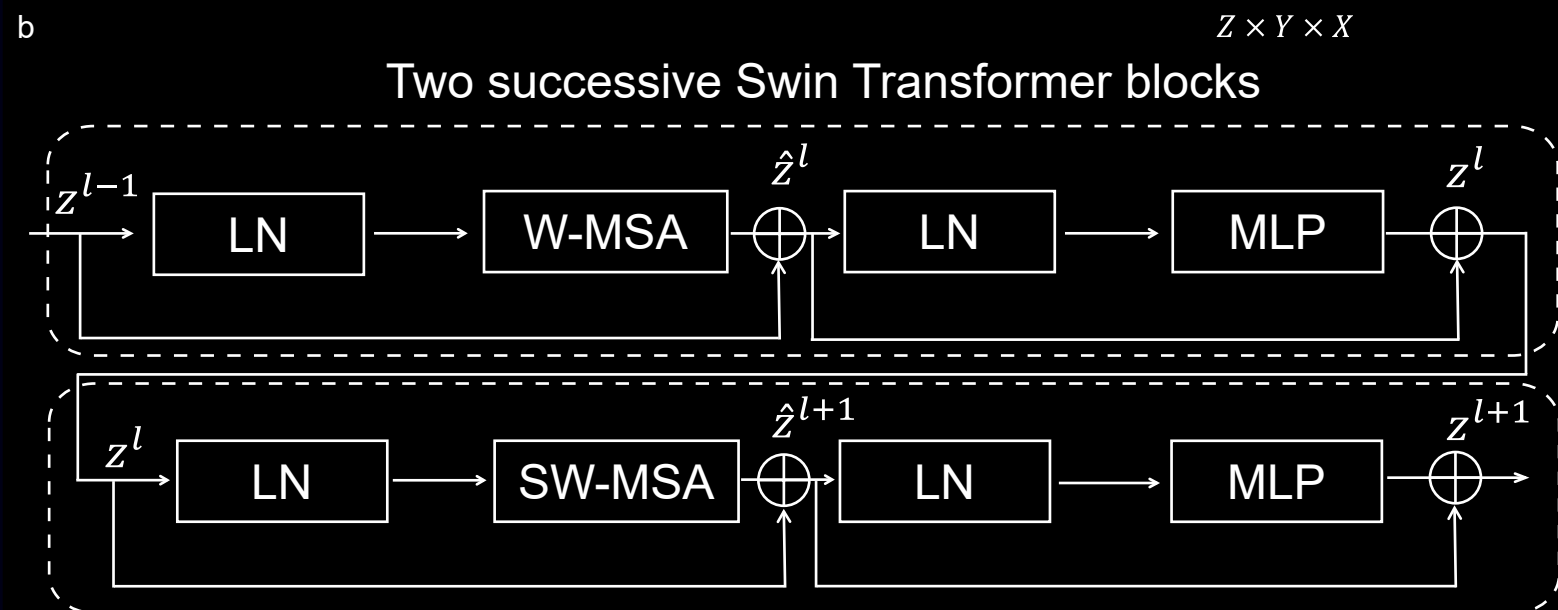

Supplement: nwae109_Supplemental_Files [file nwae109_supplemental_files.zip › Supplementary_figure_1.pdf]

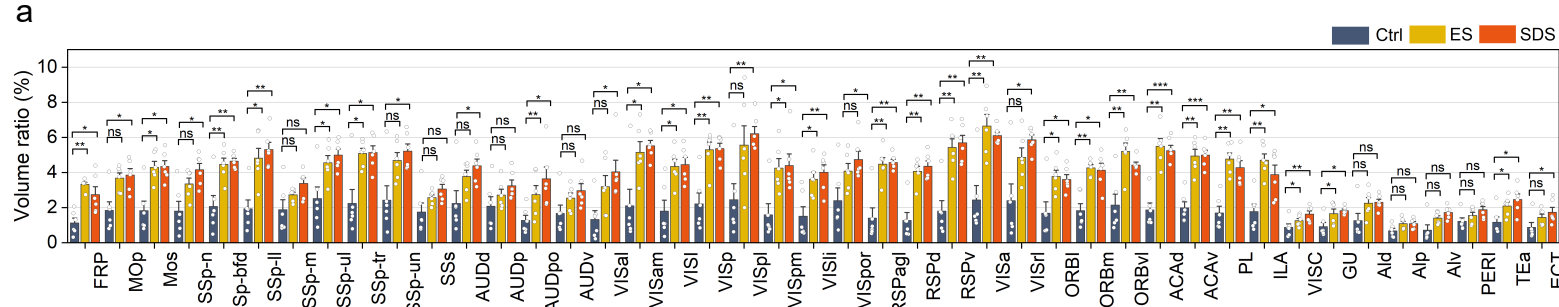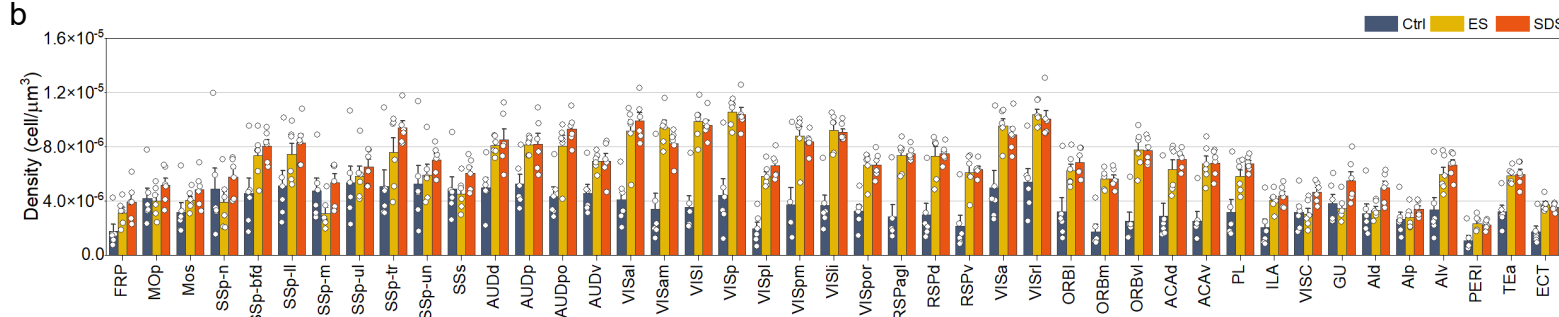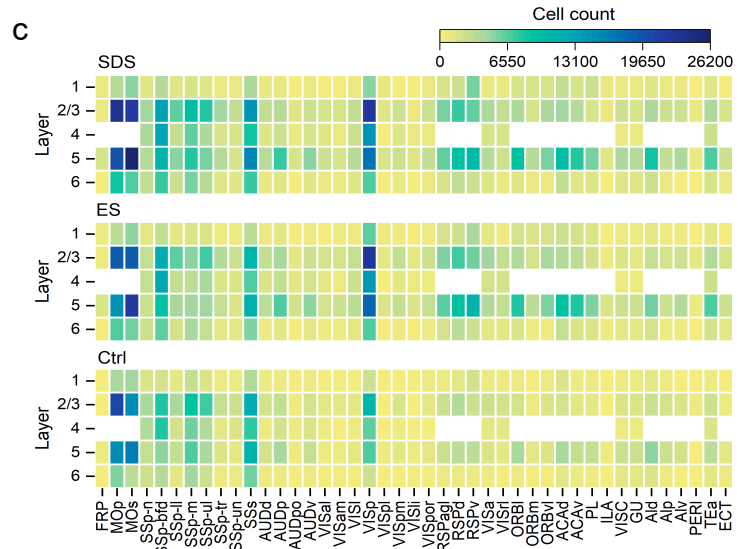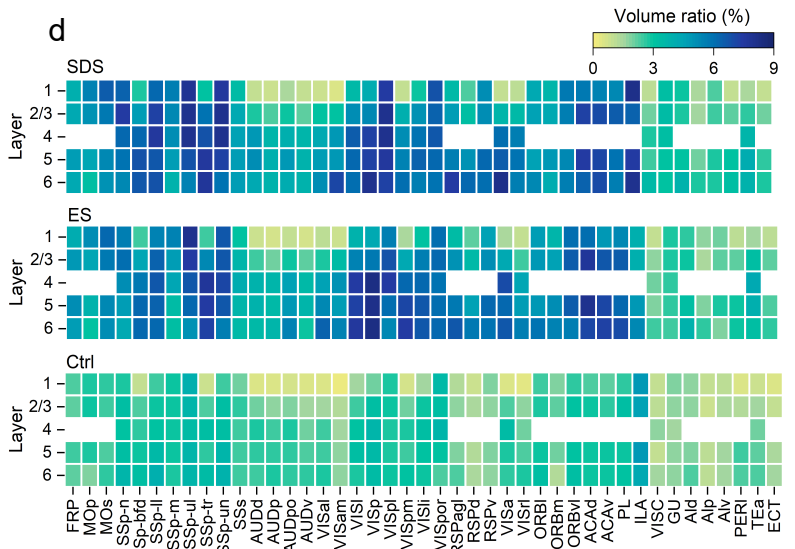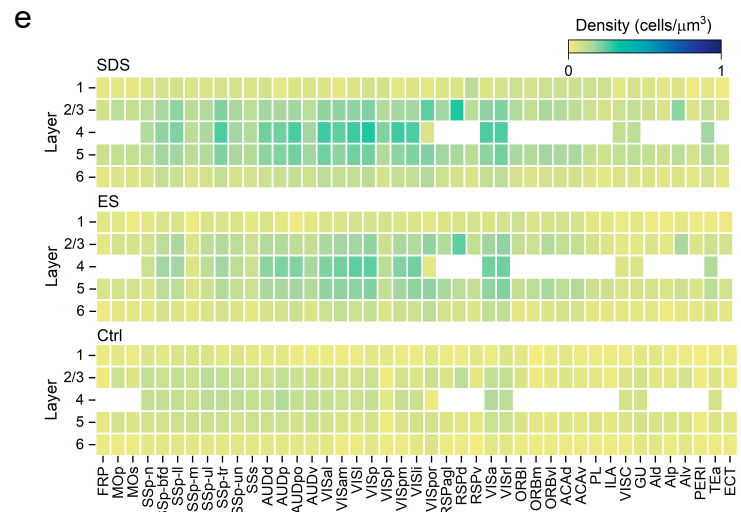

Supplement: nwae109_Supplemental_Files [file nwae109_supplemental_files.zip › Supplementary_figure_10.pdf]

Forced swimming

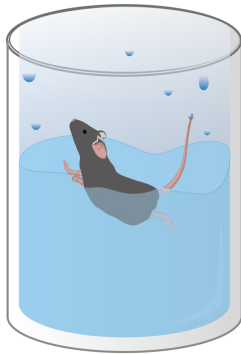

Fixation

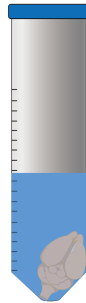

Embedding

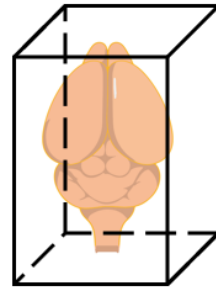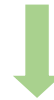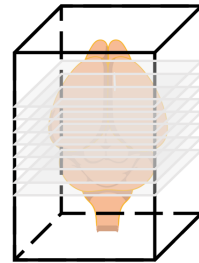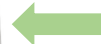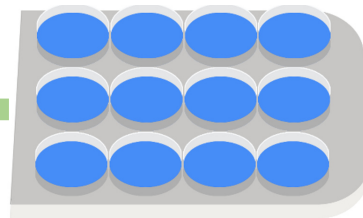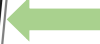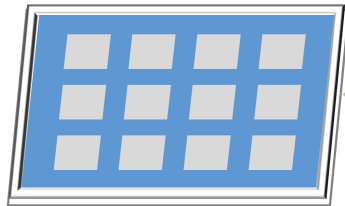

RI matching

Clearing and immunostaining

Sectioning

Supplement: nwae109_Supplemental_Files [file nwae109_supplemental_files.zip › Supplementary_figure_11.pdf]

c-Fos

2D spot filter

3D spot filter

Z=1

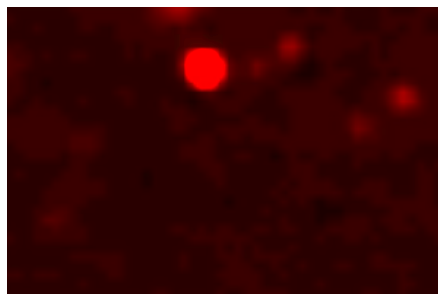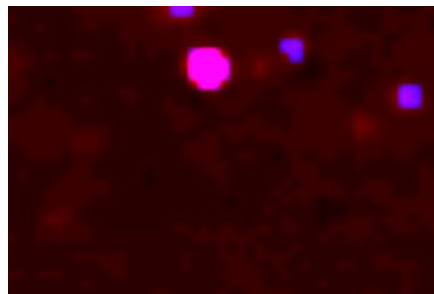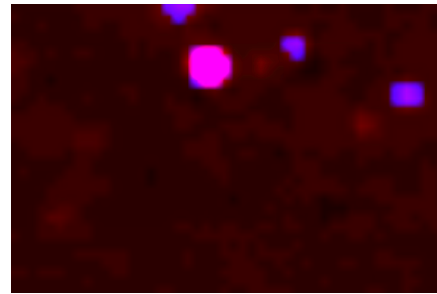

Z=2

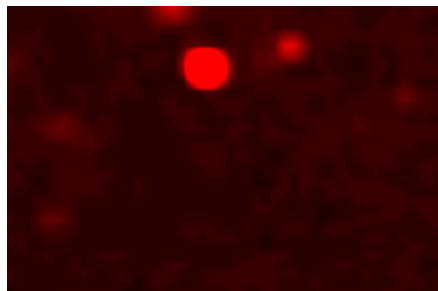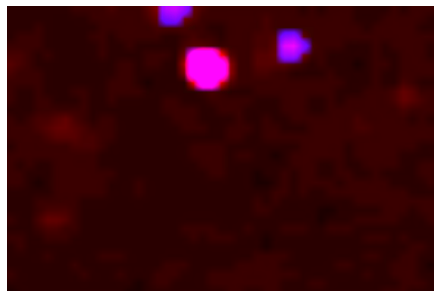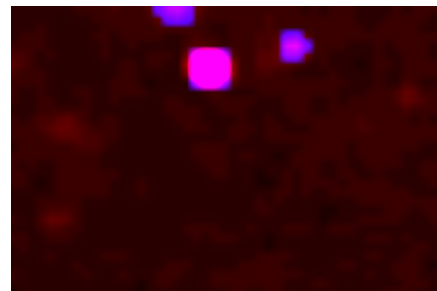

Z=3

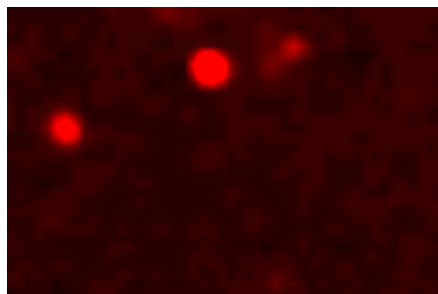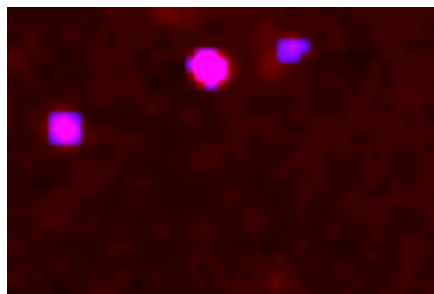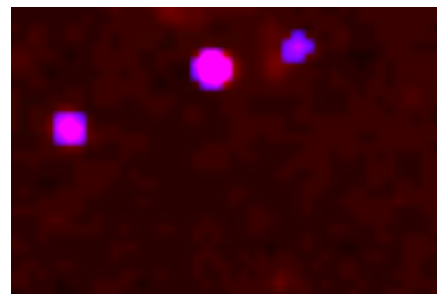

Z=4

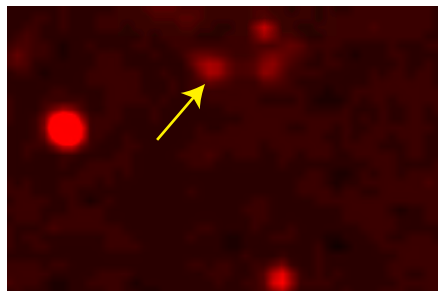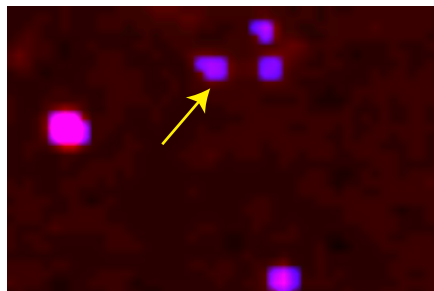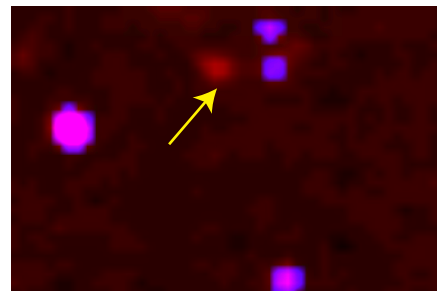

Supplement: nwae109_Supplemental_Files [file nwae109_supplemental_files.zip › Supplementary_figure_12.pdf]

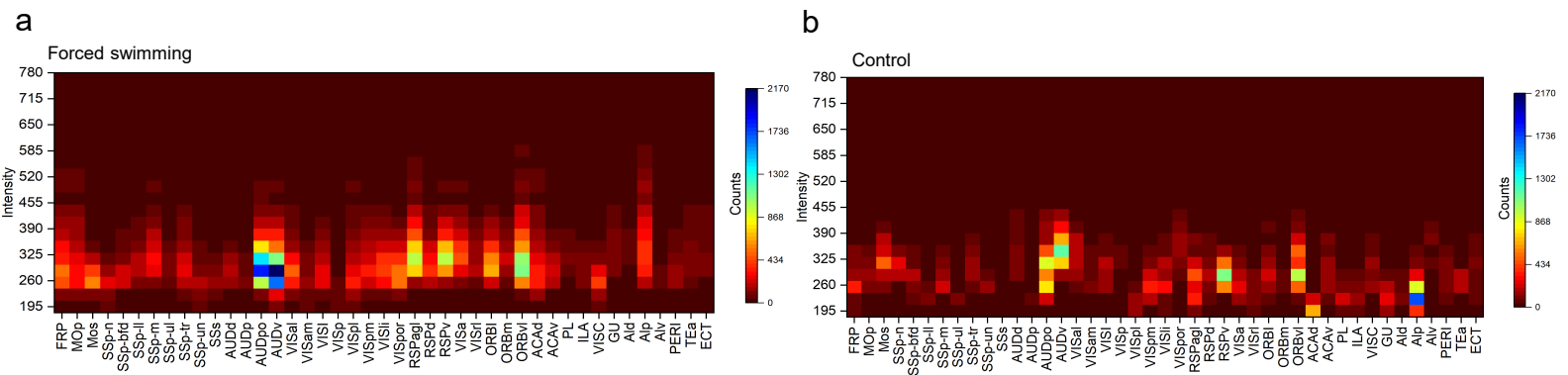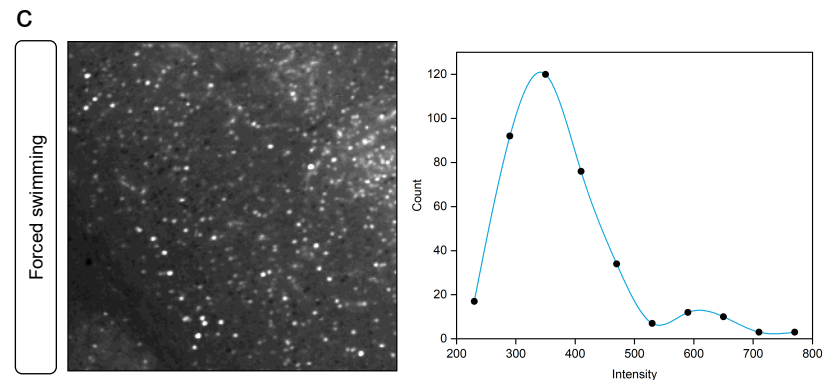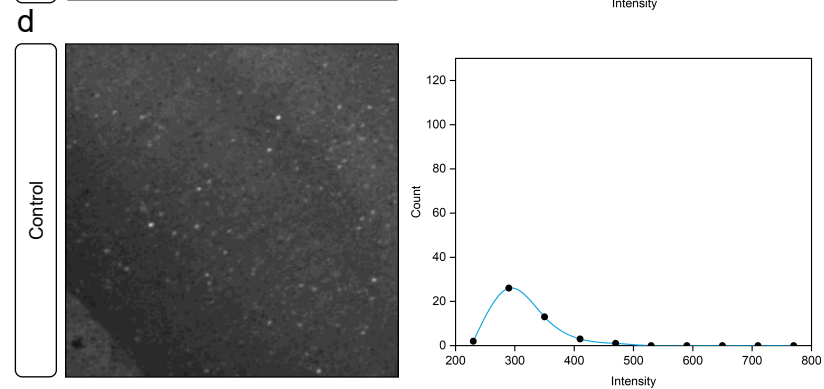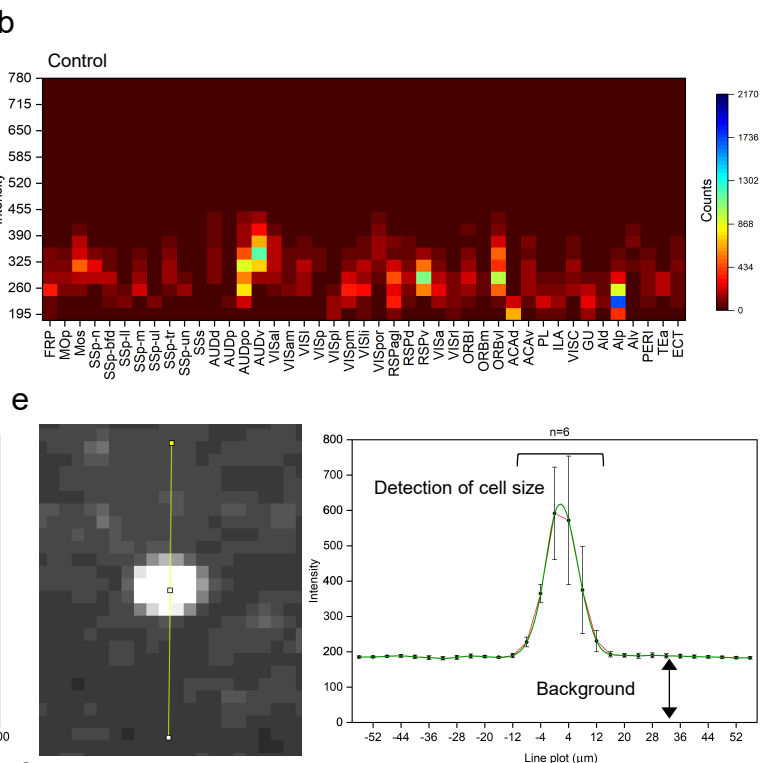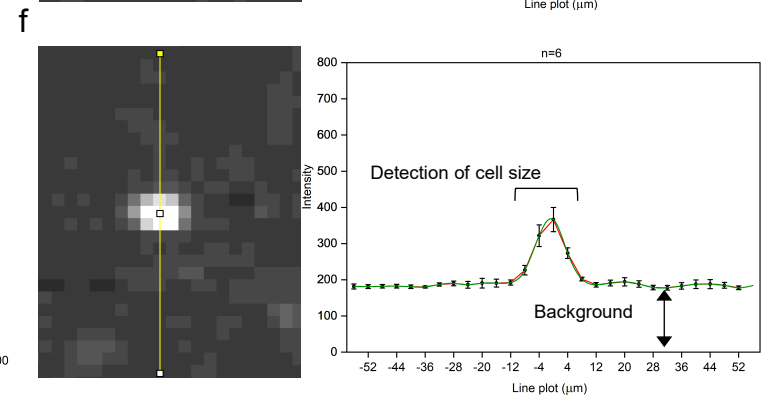

Supplement: nwae109_Supplemental_Files [file nwae109_supplemental_files.zip › Supplementary_figure_3.pdf]

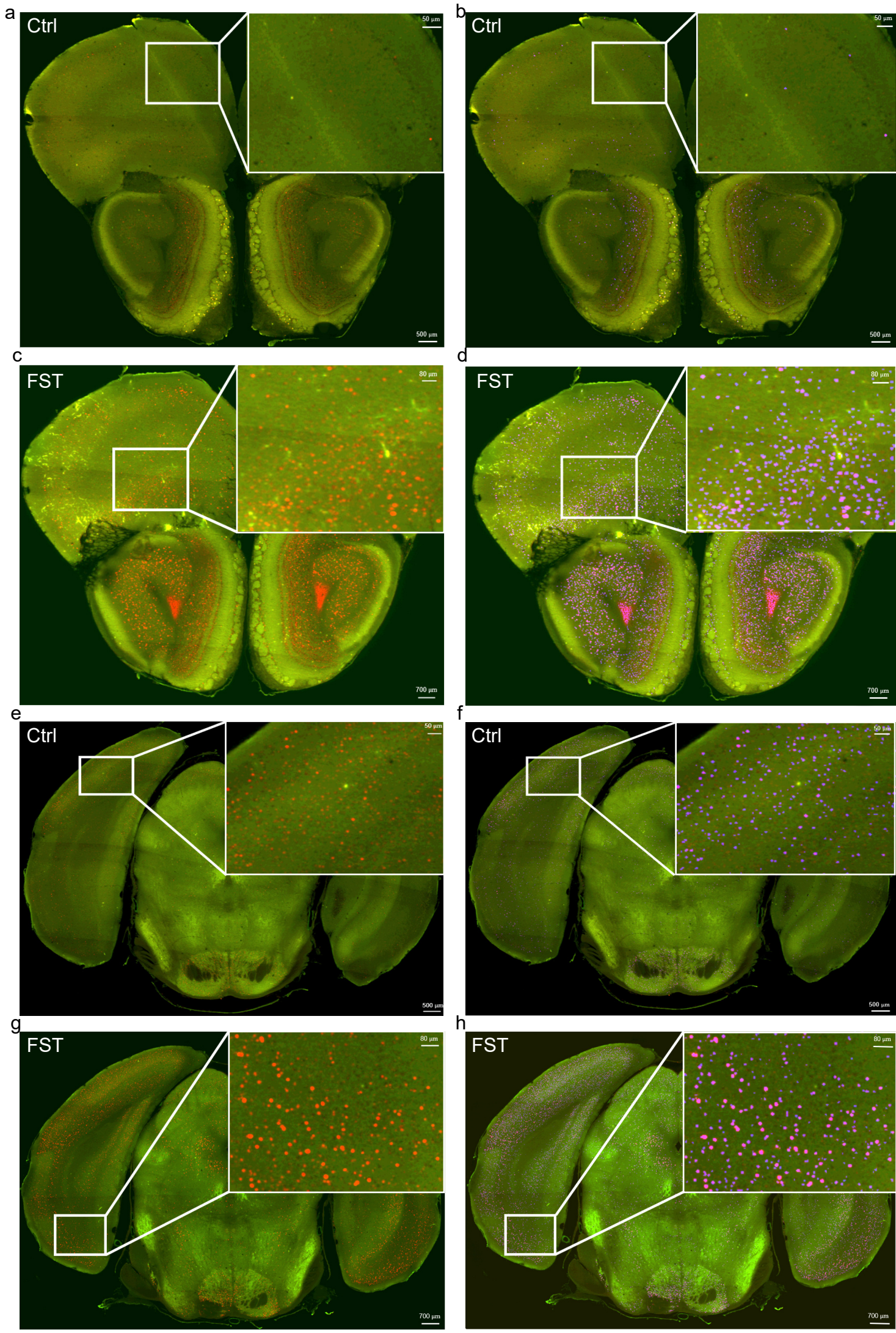

Supplement: nwae109_Supplemental_Files [file nwae109_supplemental_files.zip › Supplementary_figure_4.pdf]

a

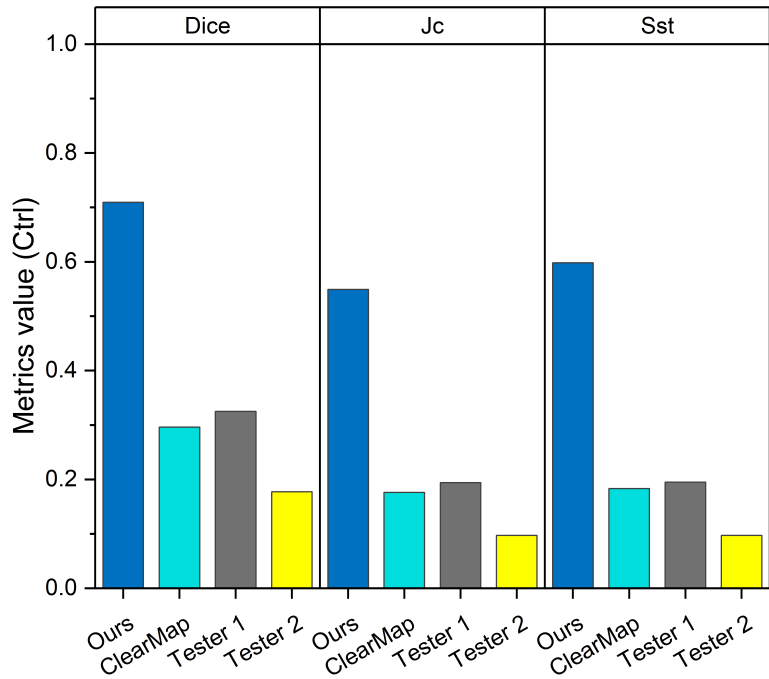

b

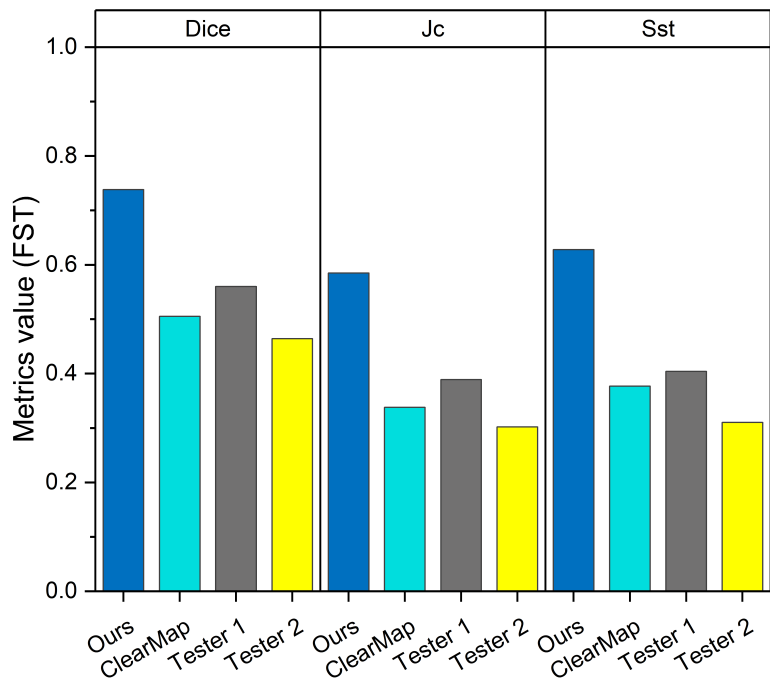

Supplement: nwae109_Supplemental_Files [file nwae109_supplemental_files.zip › Supplementary_figure_5.pdf]

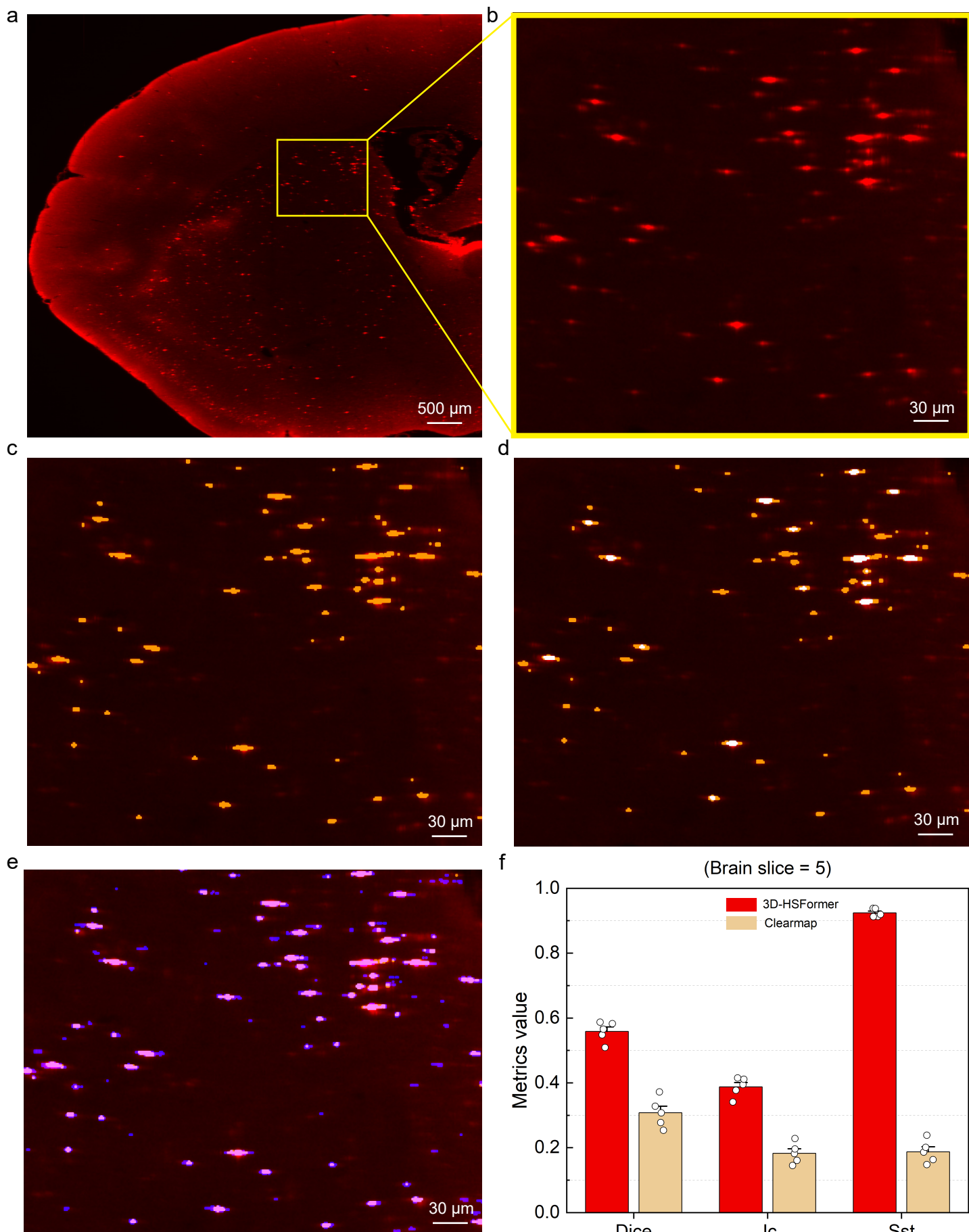

Supplement: nwae109_Supplemental_Files [file nwae109_supplemental_files.zip › Supplementary_figure_6.pdf]

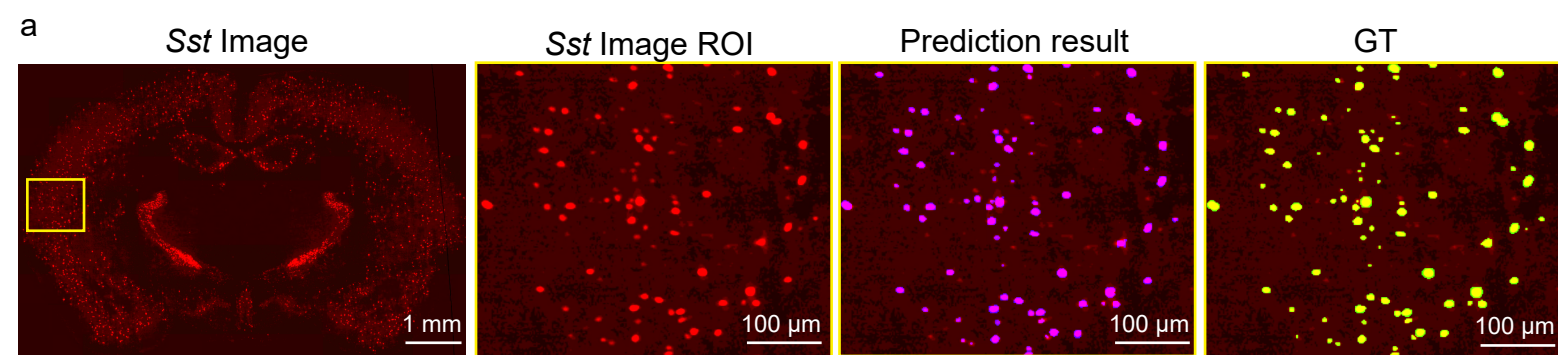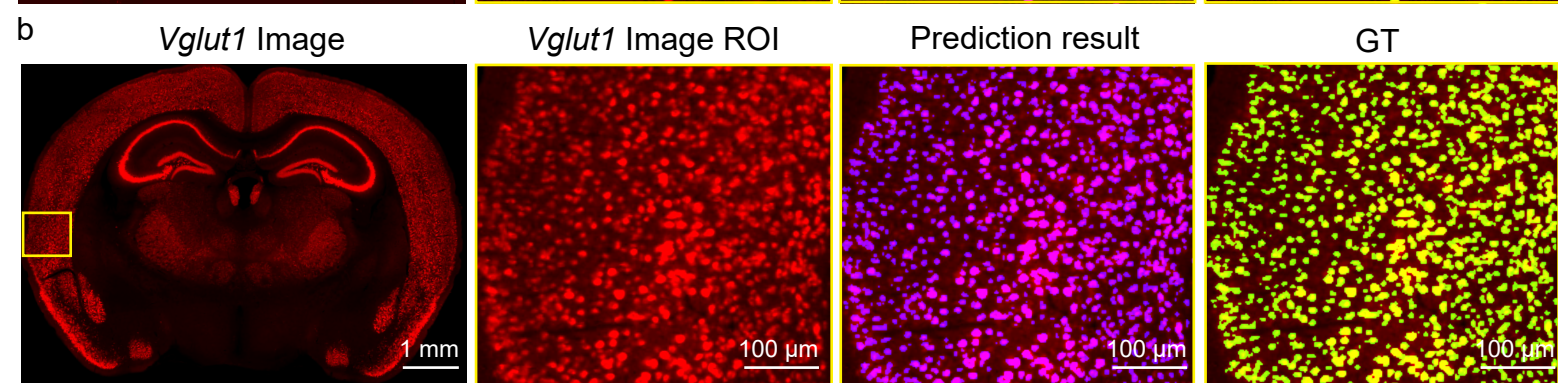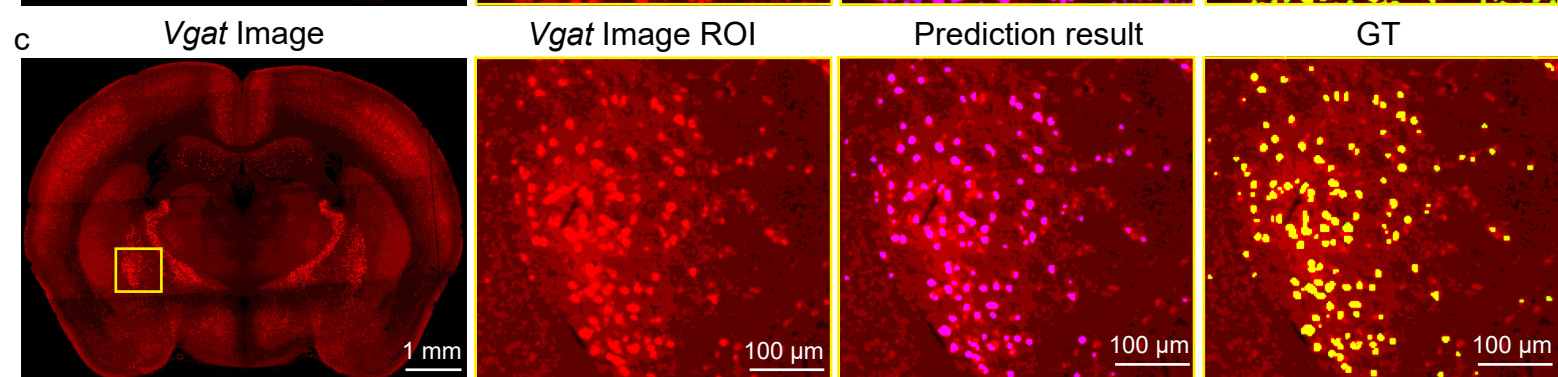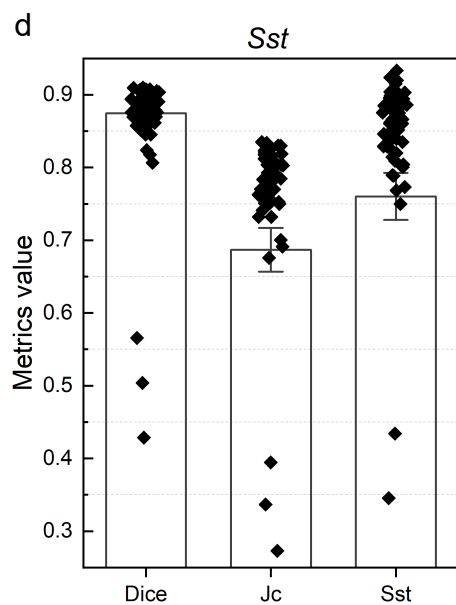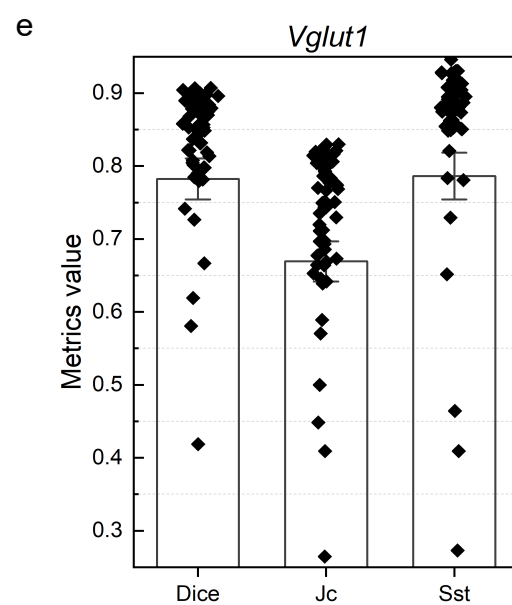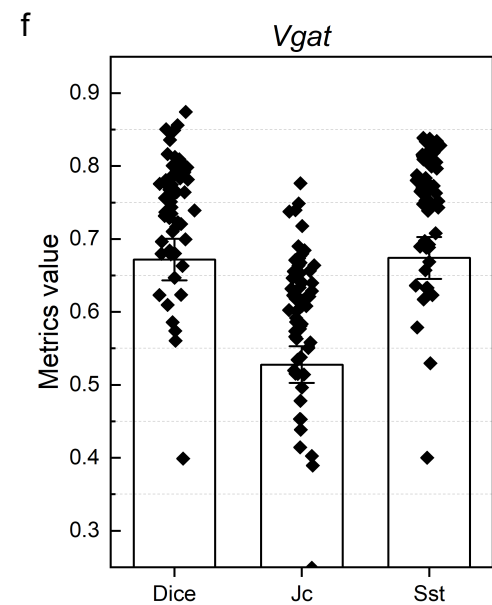

Supplement: nwae109_Supplemental_Files [file nwae109_supplemental_files.zip › Supplementary_figure_7.pdf]

a

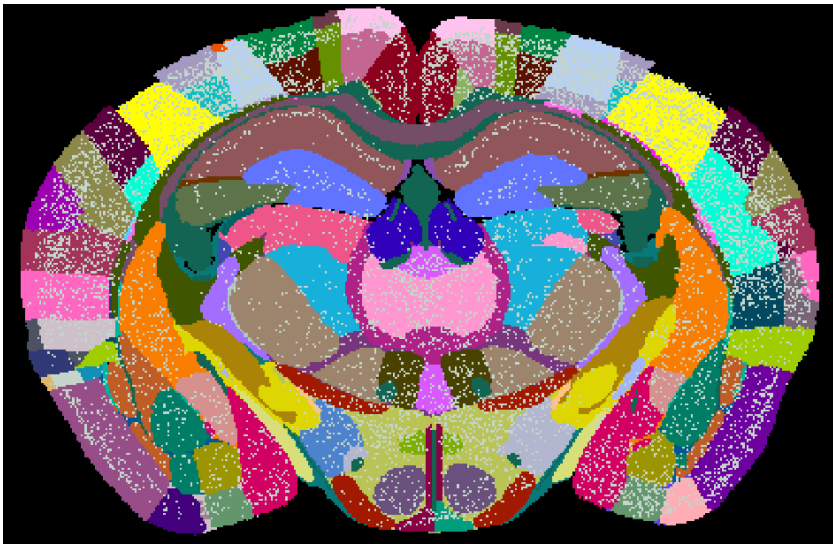

b

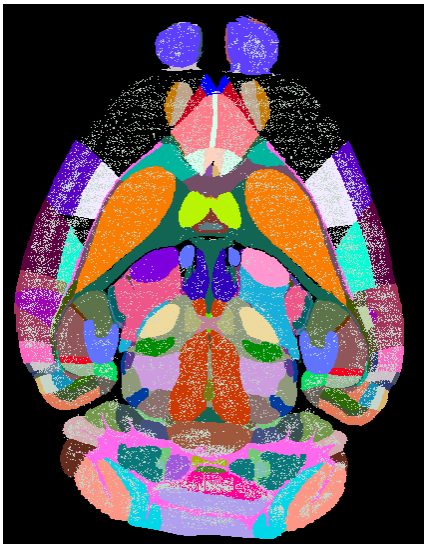

c

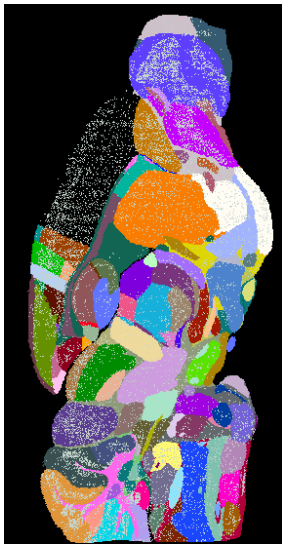

Supplement: nwae109_Supplemental_Files [file nwae109_supplemental_files.zip › Supplementary_figure_8.pdf]
